# *Lutjanus synagris* (Linnaeus 1758) age-based life history using a multi-model inference approach for growth in the southern Gulf of Mexico

**DOI:** 10.1371/journal.pone.0353946

**Published:** 2026-07-21

**Authors:** Isabel Cervantes-Camacho, Ximena Renán, Gabriela Galindo-Cortes, Teresa Colás-Marrufo, Virginia Noh-Quiñones, Thierry Brulé

**Affiliations:** 1 Departamento de Recursos del Mar, Centro de Investigación y de Estudios Avanzados del Instituto Politécnico Nacional, Yucatán, México; 2 Instituto de Ciencias Marinas y Pesquería, Universidad Veracruzana, Boca del Río Veracruz, México; University of Messina, ITALY

## Abstract

Lane snapper *Lutjanus synagris* is an important species that supports both commercial and recreational fisheries. In the southern Gulf of Mexico, lane snapper is one of the snapper species with the highest annual catch volumes. Nevertheless, information on several life-history traits such as longevity, natural mortality and age at maturity is lacking, which are important for providing appropriate management options. From the total captured lane snapper (n = 1150), 367 were used to estimate the age-life history. Specimens were captured through the small-scale fleet of Yucatan from 2008 to 2009 in southern Gulf of Mexico with total lengths from 14.50–45.90 cm and whole weights from 0.05–1.10 kg. Thin otolith sections were used to determine the age of lane snapper. Left sagittae were embedded in clear epoxy resin, thin sectioned (300 µm thickness), and analyzed using a stereomicroscope, counting opaque zones (white) deposited annually during late spring to early summer. Estimated ages ranged from 0^+^ to 16 years for females (n = 189) and from 0^+^ to 15 years for males (n = 164). In the growth modeling process, three candidate models were fitted to improve the plausibility of growth estimates under conditions where both small/young and large/old individuals are poorly represented, and the observed length-at-age data show high variability. A Bayesian approach based on the Markov Chain Monte Carlo was used, with informative priors on the growth parameters. During model fitting, three‑parameter versions were used: k, *L*_*∞*_, and a third parameter based on length-at-birth, *L*_*0*_; among these, the last two parameters have the same interpretation across all models. The best growth model by sex was selected based on the Leave-one-out cross-validation technique. The von Bertalanffy growth model was the best-fitting model for growth in both females and males. Growth parameters for females were for maximum mean length or asymptotic length (*L*_*∞*_) = 32.21 cm total length; growth coefficient (*k*_1_) = 0.27 year^-1^; size-at-age-zero (*L*_*0*_) = 2.06 cm and for males *L*_*∞*_ =  28.32 cm total length; *k*_1_ =  0.41 year^-1^; *L*_*0*_  = 2.03 cm. Natural mortality was estimated at 0.39 year^-1^ for females and 0.41 year^-1^ for males. Age at maturity in which 50% of the females and males have reached maturity was A_50_ = 3.11 years for females and 1.68 years for males. The reference points of the optimal size (L_opt_) and optimal age (A_opt_) to harvest the specimens to achieve maximum yield were 26.17 cm total length, 6.20 years for females and 21.24 cm total length, 3.38 years for males. These results on the age-based life history and growth of lane snapper are novel for the southern Gulf of Mexico population. This information forms the basis for developing appropriate management measures, as catch volumes and exploitation levels are steadily increasing.

## Introduction

Lane snapper *Lutjanus synagris* (Linnaeus 1758) is widely distributed in the western Atlantic from North Carolina to southern Brazil, including the Bahamas, the Caribbean Sea and the Gulf of Mexico (GoM) [1,2]. It is particularly abundant in the Antilles, Mexico (Campeche Bank, Yucatan), Panama and the north coast of South America [3]. This stenohaline species prefers clear waters and tolerates a wide temperature range. Juveniles inhabit areas with abundant seagrass beds, rocky bottoms, estuaries and inlets [2,4], while adults live mainly around coral reefs or on vegetated bottoms with sand [2]. Juveniles remain close to the coast due to high food availability while adults can migrate to depths > 40m for feeding and reproduction [4,5].

Iteroparous, this species can undergo many reproductive events throughout its lifetime; is considered a protracted spawner (March–August) with the formation of spawning aggregations during the breading season [3,6,7]. Larvae remain in the water column for thirty days, suggesting potential high species dispersal [8]. Adults move in response to food availability, spawning season, and environmental conditions [8]. Two genetically distinct lane snapper groups have recently been identified: one consists of populations from eastern Florida, the GoM, Honduras, and Colombia, and another of populations from Puerto Rico and Brazil, derived from oceanographic barriers such as river plumes and marine currents [8].

Lane snapper can live up to 19 years old with fast growth (k = 0.39 year^-1^) [5] and an average longevity (33 years) [2,9]. Age and growth in different parts of its distribution area have been studied using size frequency analyses [10–14]; scales [15,16]; whole otoliths [17–20]; and annuli readings in thin-sectioned otoliths [5,9,13,21–24]. Variations in environmental conditions, food availability, and fishing pressure can affect growth and demographic structure [25, 26], making it essential to determine growth parameters for each population.

Growth parameter estimation requires evaluation of different growth models to ensure its goodness of fit based on the principle of parsimony [27,28]. For Atlantic Lutjanidae, the only growth estimates based on the use of multiple models are those for northern red snapper *Lutjanus campechanus* (Poey 1860) [29], yellowtail snapper *Ocyurus chrysurus* (Bloch 1791) [30], gray snapper *Lutjanus griseus* (Linnaeus 1758) [26], and mutton snapper *Lutjanus analis* (Cuvier 1828) [31]. All growth studies of lane snapper have used the von Bertalanffy general model (VBGM). However, unexamined selection of a single model can lead to overestimation of growth parameter confidence intervals, consequently lowering estimate precision [28,32].

Lane snapper forms part of the grouper-snapper complex, which is the focus of commercial and recreational fisheries throughout its distribution area [23]. In the southern GoM, the principal target species in this complex is red grouper *Epinephelus morio* (Valenciennes 1828), the stock for which is overexploited and suffers declining catch volumes [33]. In response, snapper catches have steadily increased [34,35]. Northern red snapper, yellowtail snapper, and lane snapper are the main targets of the southern GoM snapper fishery, representing 85% of total snapper landing volume [36]. In Mexico, lane and yellowtail snappers catch volume records are categorized as “Rubia and Villajaiba.” In 2024, live catch weight for this category in the state of Yucatan, was 2107 metric tonne [37]. Approximately 40% of this volume are lane snappers caught by small-scale fleet [33,38].

Lane snapper stock health in the southern GoM was assessed in the 1980s and 1990s, when the resource was considered at near its optimal exploitation level [14,15] and was declare overfished between 1980–2019 [36]. This status raises concerns since lane snapper is on the IUCN (International Union for the Conservation of Nature) Red List as near threatened, with declining populations [39–41]. Furthermore, lane snapper biology is barely known in the southern GoM. Only reproductive life-history traits and diet composition were analysed for the species in this region [42,43]. Despite its commercial importance in the southern GoM, the only management measures in place are a fin-fish fishery access permit and required use of specific fishing gear (one longline with 250 hooks of number 10/0–12/0) [33,34].

With the aim of the filling gaps in knowledge on the age and growth of lane snapper in southern GoM, the present study analysed through a multi-model inference approach which growth model (VBGM, Gompertz, or logistic) best fitted the data set and determine if the species exhibited sexually-differentiated growth. For the southern GoM, this study is the first to determine age and growth based on the annuli counts in otolith thin sections, the most accurate methodology for quantifying age in reef fish species, particularly lane snapper [2,44]. Also, it’s the first-time age at maturity is address for the specie in southern GoM. Population demography data derived from life-history parameters such as maximum size, longevity, age at sexual maturity and natural mortality is essential for evaluating fishery stocks [45]. The results presented in this work are needed for assessing productivity (the capacity for rapid recovery of depleted stocks) and vulnerability (the potential for a stock to be impacted by fisheries), both crucial parameters when making fishery management decisions [46].

## Materials and methods

### Study area

**Fig 1:**
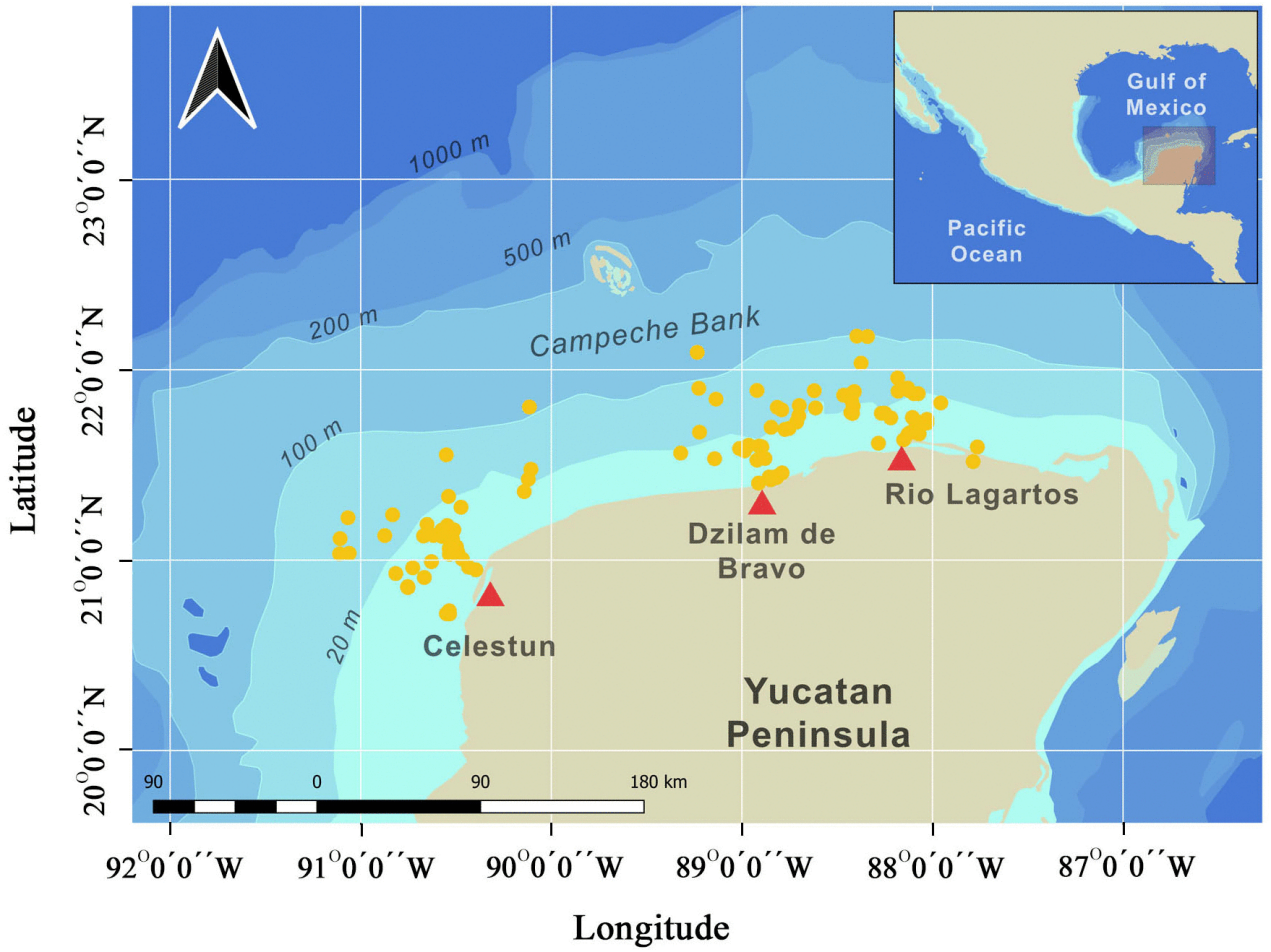
*Lutjanus synagris* sampling sites (yellow dots) in southern Gulf of Mexico during 2008–2009. Triangles indicate the three ports with the highest catch volumes of the small-scale fleet. Base map constructed using publicly available datasets from CONABIO and INEGI, including the Hypsometry and Bathymetry dataset and the State Political Division (1:4,000,000 scale). These data are distributed under a Creative Commons Attribution (CC BY 2.5 MX) license. No proprietary basemaps (e.g., Google Maps, Google Earth, or ESRI services) were used.

Lane snapper specimens were caught on the carbonate continental shelf extending west and north from the Yucatan Peninsula (20–23 °N, 92–87 °W; Fig 1) [47]. Known as Campeche Bank in the southern GoM, this area is influenced by freshwater upwelling from fractures in the karst bedrock [48]. The dominant substrate is sandy, although areas of limestone, macroalgae patches, and seagrasses are also present [49]. The Gulf Loop Current is the main marine influence in the area. It originates from the Northern Equatorial Oceanic Current which becomes the Caribbean Current and passes through the Yucatan Channel. Upon entering the Gulf of Mexico, the Loop Current makes an anticyclonic turn, exits through the Florida Strait and becomes the Florida Current, considered the initial current in the Atlantic Gulf Stream system [50]. Surface water circulation is seasonally affected by polar air masses regionally known as “nortes”, and by trade winds that, in the Northern Hemisphere, blow from northeast to southwest [51].

### Sampling

Lane snapper individuals were captured by small-scale fleet along the northern coast of Yucatan and landed in the three ports with the highest catch volumes for this species: Celestún (western region), Dzilam de Bravo (central region), and Río Lagartos (eastern region) [33,52] (Fig 1). These three ports share the same climatic tropical conditions but differ in the magnitude of the influence of the Yucatan Current, an upwelling of enriched cold waters (16.0–20.5 °C) and the different biotopes found in each site [47,48]. Lane snappers were captured from February 2008 to January 2009, using handlines or longlines with 300 hooks at 5–35 m depth, during daylight (07:00–16:00 h). Fishers held fin-fish fishing permits and complied with catch practices intended to minimize animal suffering, as per ethics guidelines in the Mexican General Wildlife Law [53]. No live animals were subjected to experimentation and the biological samples taken from each dead specimen did not affect their subsequent commercialization.

A total of 1150 individual lane snappers were processed at the landing ports. Processing involved measurement (total length = TL, fork length = FL, and standard length = SL) with an ichthyometer (± 0.1 cm precision), and weighing (whole weight = WW and gutted fish weight = GW) using a digital scale (± 0.01 kg precision). Otolith sagittae were extracted through the gill arch and placed in jars containing 70% alcohol. They were then cleaned, placed in waxed paper bags and stored in dark boxes [54]. Sagittae from each analysed individual were weighed on an analytical balance (± 0.0001 g precision) to obtain mean otolith weight (OW). Sex and sexual condition, identified by histological analysis of gonads, were taken from Trejo- Martinez et al. [42] of the same individuals in the southern GoM.

**Fig 2:**
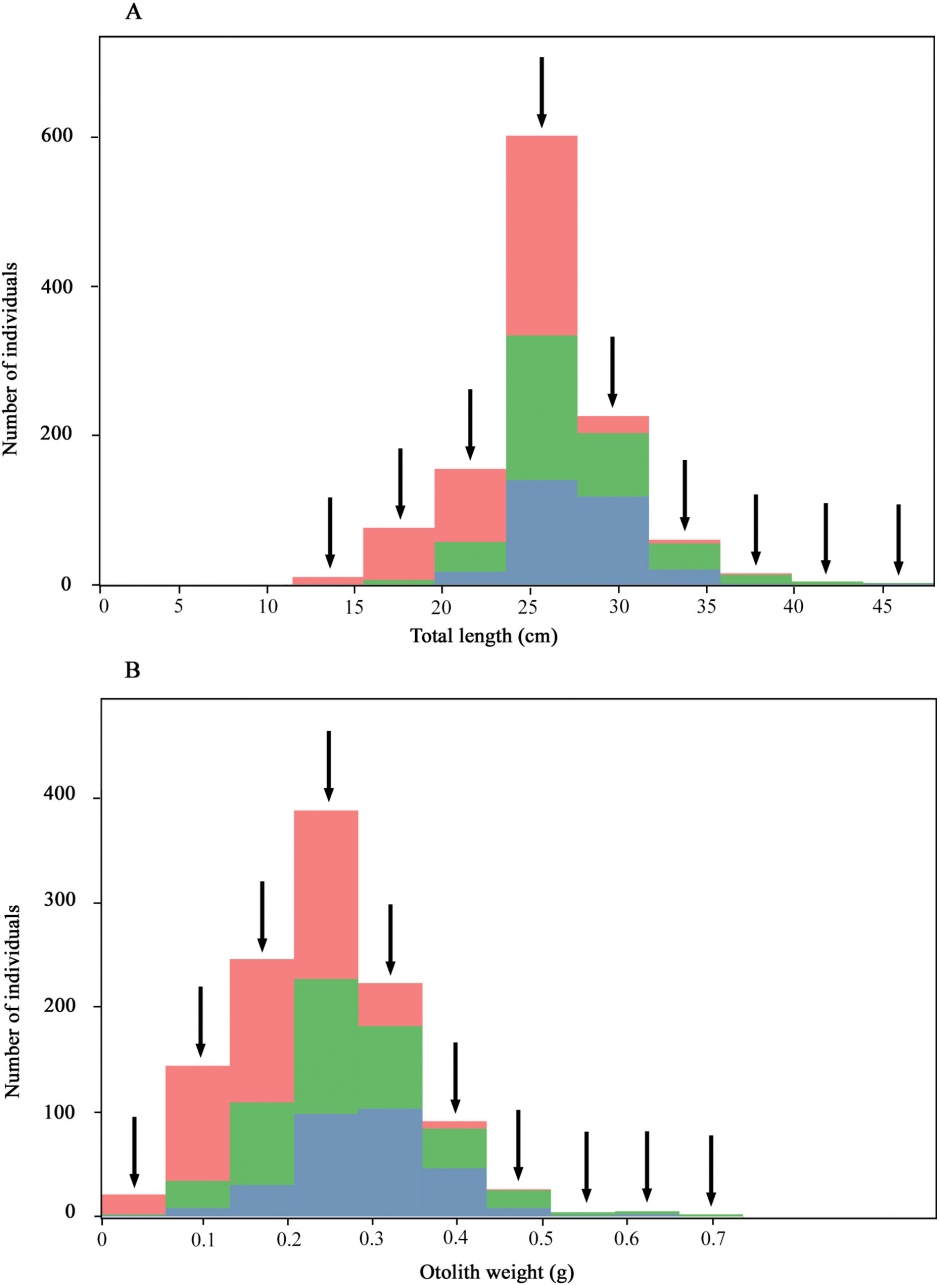
*Lutjanus synagris* total length and otolith weight frequency histograms of specimens collected in the three ports of Yucatan, in southern Gulf of Mexico, with the highest fishing landings during 2008–2009. Arrows indicate classes from which the subsample was selected and their correspondence to total length classes. Individuals in red from Celestún, in green form Dzilam de Bravo, and in blue from Río Lagartos ports, n = 1150.

To reduce time and costs in the estimation of age and growth, a subsample of otoliths was selected based on frequency histogram of the OW of all individuals (n = 1150) (Fig 2). Number of classes and class intervals of frequency histogram were generated using the Sturges’ rule [55]. For each OW established classes, 30% of the specimens were selected, without compromising accuracy and precision [18,24]. The subsample was selected taking the following into consideration: 1. Shape of TL and OW distribution, 2. sex (an equal number of male and females), 3. sexual condition (juveniles and adults), and 4. individuals from all fishing sites (Fig 2). In classes with few individuals, all specimens were analysed, with attention to smaller and large individuals, which are commonly scarce in a population, but are essential to the determination of the growth curve parameter *k* [56]. The selection of the otoliths subsample used in the estimation of the growth parameters, also followed established guidelines of 10 fish aged for each 10 mm length classes which provides a near optimal performance in accuracy and precision (Fig 2) [57,58]. To ensure a similar shape TL distribution of the all-dataset and the subsample, a kernel density estimator with a 95% confidence level was created. In addition, the representativeness of the subsample was evaluated by comparing it with the TL quantiles of the complete dataset, using 95% confidence intervals (95% CI), which were calculated using the Bootstrap method with 2000 iterations. All calculations and plots were performed using the R programming language [59].

### Otolith treatment

All left sagittae were embedded in transparent epoxy resin and cut in 300 µm-thick sections with a low-speed saw (Isomet 1000, Buehler^®^), ensuring the cut passed as near the otolith core as possible. If the left sagittae was broken or missing the right was used. The thin sections were mounted on slides with Entellan^®^ resin and viewed with a stereomicroscope using reflected light and a black background; a drop of chamomile oil was added to enhance contrast between growth zones. Under these conditions, translucent zones (TZ) – representing rapid growth – appear black, while opaque zones (OZ) – representing slow growth – appear white. An OZ + TZ pair is treated as corresponding to one annulus.

### Age validation

Calculating OZ formation period and frequency was done by marginal increment analysis (MIA), an indirect way of validating annulus deposition during an annual period. For MIA, the Age & Shape software (Infaimon) was used to measure the distances from the otolith core to the last two OZs and to the edge (radius). The marginal increment (MI) was calculated as: *MI = (R – r*_*i*_*)/ (r*_*i*_ *– r*_*i-1*_*)* where R is the radius, *r*_*i*_ is the distance from the core to the last OZ, and *r*_*i-1*_ is the distance from the core to the penultimate OZ [60]. After calculating the individual MI ratios, these were averaged by month of capture over a one-year period and graphed together with an annual analysis of zone type (OZ or TZ) observed at the otolith edge. In an effort to limit measurement errors from use of a generalized analysis of annual increment [61], and considering that the MIA is better suited to young individuals [62], these analyses were run only using otoliths displaying 3–9 OZ [22]. Calculations were also done of MI by each OZ groups, and by sex, analysing monthly variations in OZ period of deposition over an annual cycle.

### Age determination

Observed age was measured by counting the annuli from otolith core to edge at the ventral apex and along the margin of the sulcus acusticus [62]. The first opaque mark out from the otolith centre was discarded from the count because it corresponds to a false ring reported for the family Lutjanidae [62,63]. Annuli counts were done by two independent readers and using the Age & Shape program (Infaimon), which automatically identifies growth zones. Counts were done with no prior knowledge of an individual’s size, OW, or sex. When inter-reader discrepancies in annuli count arose, the otolith in question was recounted by the most experienced reader until reaching consensus. If a discrepancy persisted after the recount, the sample was eliminated from the study. Annuli count accuracy was estimated by calculating the overall average percentage error (APE), APE between readers, APE for the automatic count (Age & Shape), APE for age groups (3 years–9 years), and the general coefficient of variation (CV), taking into account the landmark of 5.5% APE and 7.6% CV established for otolith readings [61,64,65].

Biological age or fractional age was calculated by adding the fraction of time elapsed between each individual’s theoretical birth date and capture date to the observed age [54,66]. The theoretical birth date was June 16, the halfway point of lane snapper’s spawning season (May–July) in the southern GoM [42]. For individuals captured during the OZ formation period, as derived from the MI results, but which had not yet formed an OZ, one year was added to the biological age, considering that the individual was close to attaining one more year of life [67].

### Growth parameterization

Growth models are sensitive to the absence of small juveniles or large adults, which leads to the estimation of biologically unreasonable parameters [45,68]. To address this, three asymptotic growth models were fitted to length at age data for lane snapper, explicitly incorporating prior knowledge of the model parameters in a Bayesian framework to improve the estimates [68,69]. Parameterizations based on size-at-age zero (*L*_*0*_) were preferred, under the assumption that this is a better descriptor of the theoretical mean length at age zero [68–70].


$$\begin{array}{c} \text{Traditional von Bertalanffy model }(\text{VBGM}):L_{\left(t\right)}=L_{\infty }-\left(L_{\infty }-L_{\text{0}}\right)e^{k\text{1}t} \end{array}$$ (1)

$$\begin{array}{c} \text{Gompertz model}:\;L_{\left(t\right)}=L_{\text{0}}e^{\left(log\left(L_{\infty }/L_{\text{0}}\right)\left(\text{1}-e^{-k\text{2}t}\right)\right)} \end{array}$$ (2)

$$\begin{array}{c} \text{Logistic model}:{\;L}_{\left(t\right)}=\left(L_{\infty }L_{\text{0}}e^{k\text{3}t}\right)/\left(L_{\infty }+L_{\text{0}}\left(e^{k\text{3}t}-\text{1}\right)\right) \end{array}$$ (3)

For all models $L_{\left(t\right)}$ is mean length at age *t* (TL in cm), *L*_*∞*_ is maximum mean length, and $L_{\text{0}}$ is length-at-age zero (in cm). In the VBGM (1) *k*_*1*_ (in year^-1^) is the rate at which growth approaches this asymptote such that it takes ${ln\;\text{2}\;k}_{\text{1}}^{-\text{1}}$ units of time to grow halfway towards *L*_*∞*_ at any given point [71]. In the Gompertz growth model (2) *k*_*2*_ is the rate of exponential decrease of the relative growth rate with age and in the logistic growth model (3) *k*_*3*_ is the relative growth rate parameter [28].

In this study, we use the approach proposed by Smart and Grammer [69] to fit Bayesian growth models using the Markov Chain Monte Carlo (MCMC) method, with prior information for the growth parameters (*L*_*∞*_, *L*_*0*_*,* and *k*) to improve the biological plausibility of the growth estimates. We used the generalized framework implemented in the R package ‘BayesGrowth’ [72] where each growth parameter requires a prior distribution. Normally distributed informative priors were set for *L*_*∞*_ and *L*_*0*_, with mean *L*_*∞*_ initially deﬁned from the maximum length of ﬁsh observed (45.9 cm TL) and *L*_*0*_ set to 2 cm TL based on reported length-at-birth for lane snapper [73]. Standard deviation was defined as 10% of the mean value for *L*_*∞*_ and as 0.1 for *L*_*0*_ [68]. Meanwhile, the remaining parameters (*k*) and the residual standard error (σ) had a uniform distribution bounded between zero and a maximum probable value (2 year^-1^ and 5 year^-1^, respectively). Four MCMC chains with 10,000 simulations were used to determine parameter posterior distributions, of which 5,000 were discarded after the burn-in phase and thinning = 1 [69,72]. Each Bayesian model was evaluated for convergence and efficiency using the R-hat statistic and the Effective Sample Size generated by the ‘rstan’ R package [74]. In addition, diagnostic plots generated with the ‘Bayesplot’ R package [75] were examined to confirm that the chains were well-mixed and free of autocorrelation (S1 Fig).

The ‘BayesGrowth’ package allows for the estimation of growth models using MCMC by specifying informative priors based on known *L*_*0*_ and *L*_*∞*_, and also provides credibility intervals around the growth curves. Within a multiple-model approach, Bayesian model selection is performed using leave-one-out-cross-validation (LOOCV), currently considered one of the most robust methods for evaluating out-of-sample predictive accuracy based on the log-likelihood calculated over the posterior distributions of the parameters [69]. From LOOCV, we obtain the leave-one-out information criterion (LOOIC), analogous to the Akaike information criterion (AIC) in the frequentist approach, as well as the LOOIC weights (LOOICw) for each candidate model, which are interpreted equivalently to the AIC weights in model selection [27,28,69].

For the VBGM model, the growth performance index (Phi prime; $\emptyset^{\prime }$) was calculated to compare the growth parameters used here with previously published parameters for the same species. Phi prime was calculated from the asymptotic length ($L_{\infty }$; TL cm) and the growth coefficient (*k*_*1*_; year^-1^), with the formula: $\emptyset^{\prime }=logk_{\text{1}}+\text{2}log\left(L_{\infty }\right)$ [76].

### Life history

Natural mortality was calculated from the VBGM growth parameters. Overall natural mortality (M_overall_) was calculated from: *M*_*overall*_ *= 4.899*×*Amax*^*-0.916*^ [77] and mortality-at-age (M) was calculated using the equation: $ln(M)=\text{0}.\text{5}\text{5}-\text{1}.\text{6}\text{1}ln(TL)+\text{1}.\text{4}\text{4}ln\left(L_{\infty }\right)+ln\left(k_{\text{1}}\right)$ [78].

Age at sexual maturity (A_50_), or the age at which 50% of the individuals have reached maturity, was calculated using generalized linear models with a binomial logit link function (0 immature, 1 mature). The 95% CI were estimated using the Bootstrap method with 1000 iterations. In both cases, the ‘plot maturity’ function of the ‘ggFishPlots’ package, the R program was used [21,59,79].

Finally, the reference points of the optimal size (*L*_*opt*_) and optimal age (*A*_*opt*_) at which specimens should be harvest in order to reach the maximum yield and revenue, were calculated from the VBGM growth parameters [80]. For optimal size *L*_*opt*_ *= L*_*∞*_ × *(3/(3 + M/k*_*1*_*))* and the corresponding optimal age, estimated by applying the inverse equation of the VBGM: *A*_*opt*_ *= t*_*0*_*–1/k*_*1*_ × *ln(1 – L*_*opt*_*/L*_*∞*_*).* Similarly to sexual maturity, female and male size at maturity values (*L*_*50*_) used in the present study were also obtained from Trejo-Martinez et al. [42].

## Results

### Sampled lane snappers

**Fig 3:**
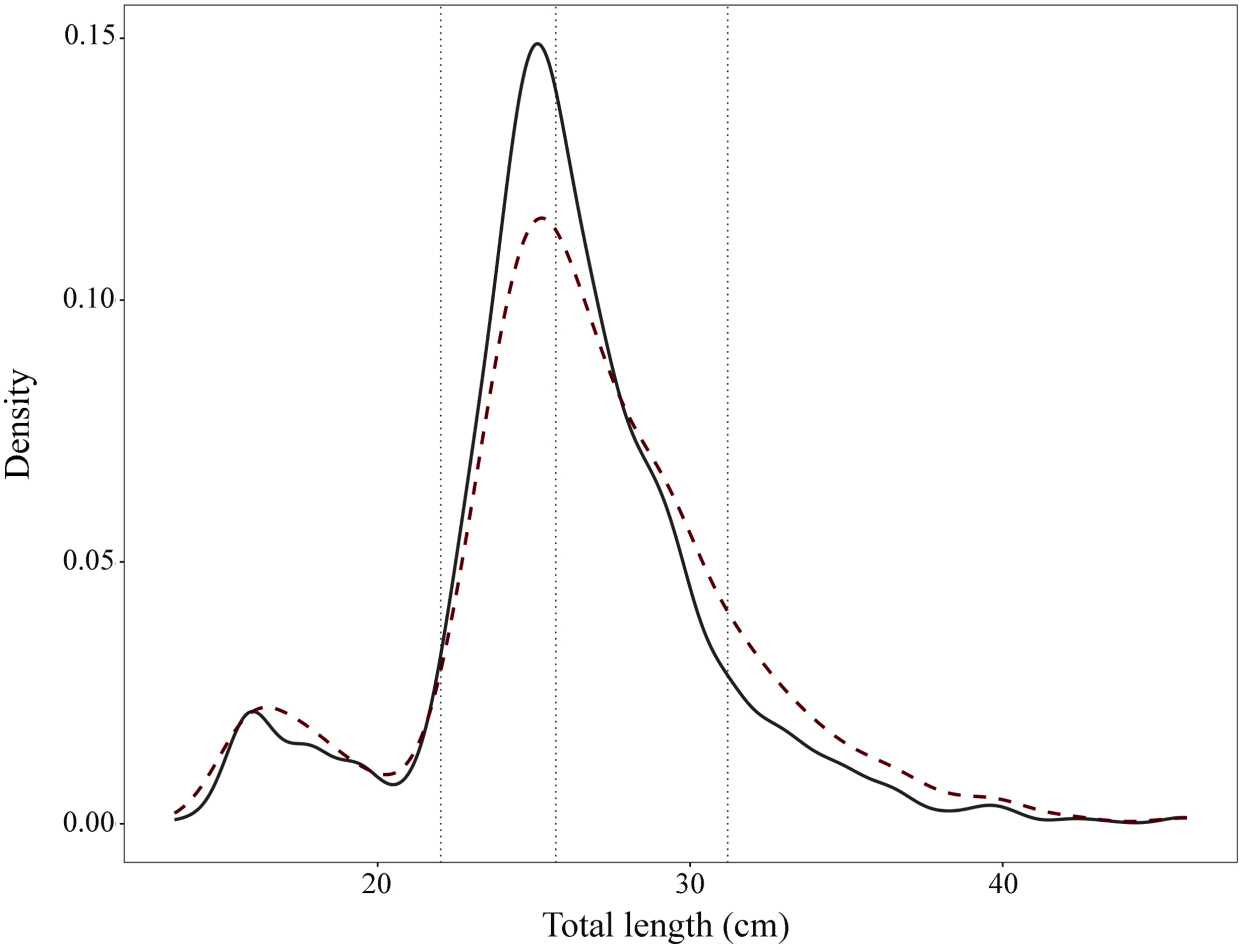
Total length Kernel density plot of the subsample and all-dataset individuals of *Lutjanus synagris* captured in southern Gulf of Mexico during 2008–2009. All-dataset (grey continuous line), subsample (red dashed line), and percentiles of all-dataset (dotted vertical lines).

Fish size (TL) ranged from 14.50 to 45.90 cm, with mean of 25.90 cm SD = 4.20 cm (n = 1150). Individuals of the otoliths selected for age determination (n = 367), named subsample, varied in size from 14.50 to 45.90 cm, with mean of 26.60 cm SD = 4.90, demonstrating that all size classes obtained in the catches were included in the age estimations. The same was true for OW where the total of otolith samples (0.02–0.70 g OW; mean 0.24 g SD = 0.10 g) and the selected subsample (0.02–0.70 g OW; mean 0.27g SD = 0.13) share the same range. The Kernel density distribution indicated that subsample adequately represented the size spectrum of capture data (Fig 3). In addition, the 95% CI of the differences in quantiles between the subsample and the full dataset overlapped at zero at lower and intermediate quantiles (Q10 – Q50), suggesting not detectable size-dependent bias in these ranges. However, confidence intervals at higher quantiles (Q75 – Q90) did not overlap zero (Q75 = 0.40–1.60; Q90 = 0.20–2.50), indicating greater variability at the upper end of the size distribution rather than a consistent bias (S2 Data, S3 Fig).

Age could be determined for 353 individuals; the sagittae of fourteen individuals were discarded due to illegibility. In total 82% of the analyzed otoliths were left and 18% were right sagittae. Total length, WW and OW for these individuals ranged from 14.50–45.90 cm TL, 0.05–1.10 kg WW and 0.02–0.70 g OW for females (n = 189), and 15.40–36.50 cm TL, 0.05–0.54 kg WW and 0.03–0.61 g OW for males (n = 164), because males were smaller than females. About 11% of females were immature (n = 21) and 89% mature (n = 168), while 5% of males were immature (n = 9) and 95% mature (n = 155).

### Age validation

**Fig 4:**
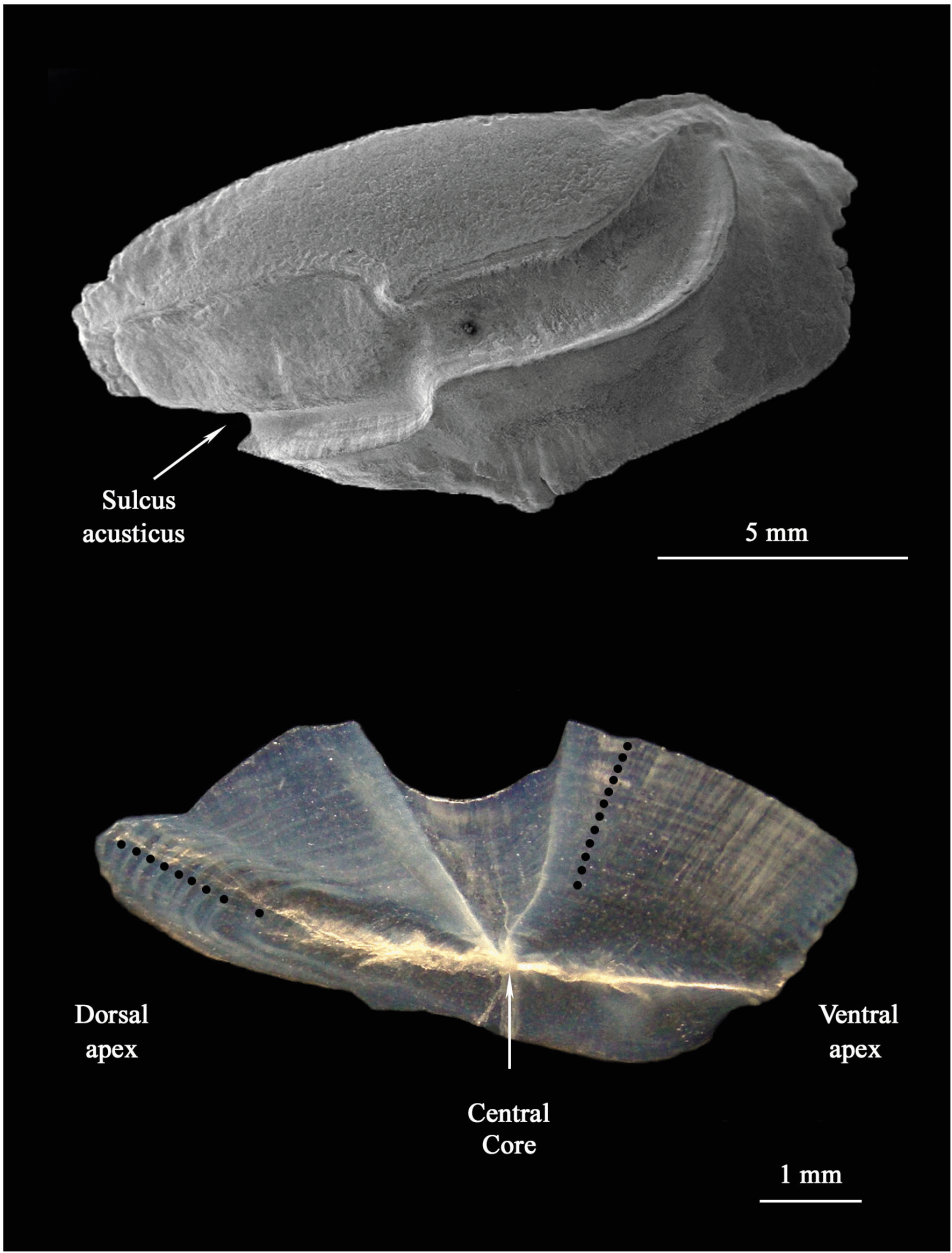
Left sagitta (above) and its thin section (below) from a male *Lutjanus synagris* captured in Río Lagartos, in southern Gulf of Mexico, on October 2008 (26.8 cm TL). Annuli (dark dots) consist of a bright opaque zone (OZ) and a dark translucent zone (TZ), which represent seasonal increments. The thin section shows a differential pattern depending on the apex*,* with 9 visible annuli in the dorsal apex and 12 in the ventral apex.

The OZ counts showed a difference in the number of OZs along the dorsal apex and those along the ventral apex. Counts were more discernible along the ventral apex (Fig 4), so it was decided to make the counts along this part of the otolith. Growth rings were identifiable as a succession of OZs and TZs extending outward concentrically from the otolith core to the edge. Distances between successive OZs decreased gradually to the edge.

**Fig 5:**
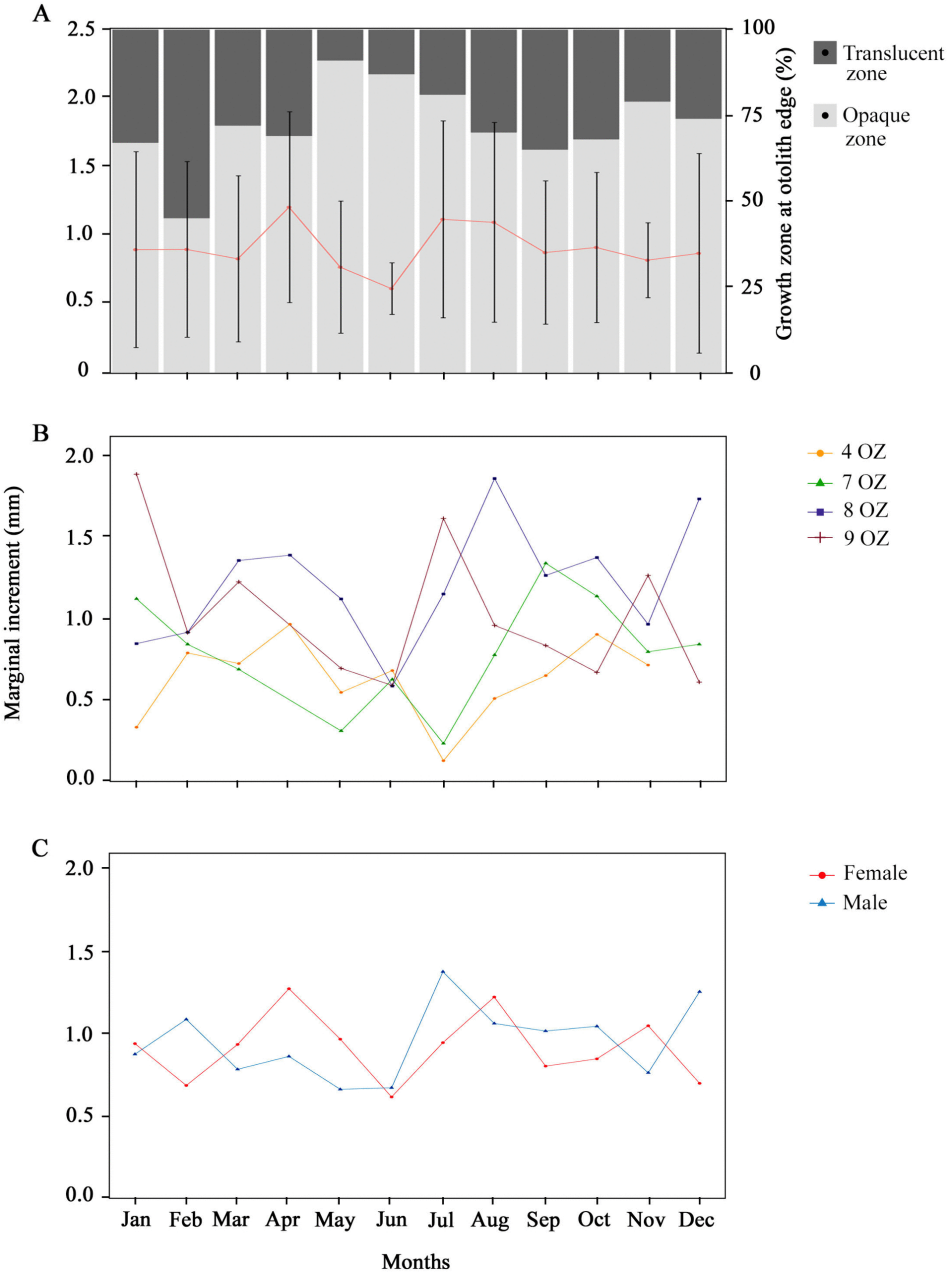
Monthly mean marginal increment in otoliths of *Lutjanus synagris* captured in southern Gulf of Mexico during 2008–2009. A– Global mean (red line ± standard deviation) with the percentage of individuals showing opaque zones (OZ) and translucent zones (TZ) at the otolith edge per month (bars). B– By group according to the number of opaque zones. C– By sex.

Average MI values were lowest in May ($\overset{―}{\text{x}}$ = 0.76, S.D. ± 0.48 mm; n = 30) and June ($\overset{―}{\text{x}}$ = 0.11 ± 0.18 mm; n = 13), suggesting that OZ formation generally occurred in these months. These same months had the highest percentage of individuals with an OZ on the otolith edge (91% in May, 87% in June). These results indicate that one OZ might be deposited annually during these months (Fig 5A).

Considering that the periodicity and frequency of OZ deposition may change in each age group or cohort, MIA were also performed separately for otoliths with 4, 7, 8, and 9 OZs (n = 139 individuals); these were the only groups present in all months of the analysed annual cycle (Fig 5A). Individuals with 4 OZs (n = 44) deposited OZs between July (MI_4OZ_ = 0.12 mm; n = 1) and August (MI_4OZ_ = 0.50 ± 0.21 mm; n = 5). Individuals with 7 OZs (n = 28) exhibited the lowest MI values from May (MI_7OZ_ = 0.30 mm; n = 1) to July (MI_7OZ_ = 0.22 mm; n = 1). Those with 8 OZs (n = 34) and 9 OZs (n = 33) deposited them in June (MI_8OZ_ = 0.57 mm; n = 1, and MI_9OZ_ = 0.58 ± 0.22 mm; n = 4) (Fig 5B). Caution should be taken as some of these values were obtained based on only one individual. Even though results show the existence of variations by age groups the periodicity of the OZ formation is consistent with the general pattern of an annual deposition.

Opaque zone (OZ) deposition might occur a month earlier in males (n = 111) in May (MI_males_ = 0.64 ± 0.39 mm; n = 9) than in females (n = 115) that deposited the OZ in June (MI_females_ = 0.60 ± 0.18 mm; n = 7). Nonetheless, both sexes always formed an OZ once a year between late spring and early summer (Fig 5C).

### Age determination

For females ages ranged from 0^+^ to 16 years (16.40–45.90 cm TL) and for males 0^+^ to 15 years (15.70–36.50 cm TL). The most common ages for females where 4–9 years (14.50–39.20 cm TL; n = 107), and for males age 3–8 years (15.40–30.10 cm TL; n = 100). The least frequent ages for females and males where those of the youngest (0^+^ and 1 years) (n = 16) and the oldest (15–16 years) (n = 20) (Table 1). Overall APE was 4.19%, APE between readers was 4.38%, and automatic count APE was 4.02%. The APE per age group declined steadily from 5.63% in 3-year-olds to 1.72% in 16-year-olds. The length-at-age keys for lane snapper exhibited wide variation in size with age, primarily among 4-year-olds (16.20–30.60 cm TL; $\overset{―}{\text{x}}$ ± SD = 22.80 ± 5.70 cm TL), 9-year-olds (23.40–37.80 cm TL; 30.20 ± 5.70 cm TL), 10-year-olds and 12-years-old (23.40–41.40 cm TL; 31.80 ± 6.70 cm TL) (Table 1). The OW-at-age keys displayed the same demographic structure than the length-at-age keys corroborating that the subsample based on the OW for age estimation was correct (Table 1). Moreover, both length – age (TL = −2.29 + 8.96 × Age) (S4A Fig) and OW – age (OW = 1.30 + 2.18 × Age) (S4B Fig) exhibited positive linear relationships, but the latter relationship had a better goodness of fit (TL – Age r^2^ = 51.44% vs. OW – Age r^2^ = 70.13%) explaining age.

**Table 1:** Age-size and age-otolith weight keys for *Lutjanus synagris* captured in southern Gulf of Mexico during 2008–2009.

Total length			Age (years)																	
Ll	Ul	Midpoint	n	0^+^	1	2	3	4	5	6	7	8	9	10	11	12	13	14	15	16
14.5	18.0	16.2	28	2	4	10	6	6	0	0	0	0	0	0	0	0	0	0	0	0
18.1	21.6	19.8	10	0	2	2	2	4	0	0	0	0	0	0	0	0	0	0	0	0
21.7	25.2	23.4	97	0	0	0	12	19	17	16	8	7	7	3	3	2	3	0	0	0
25.3	28.8	27.0	118	0	0	2	4	16	15	6	14	15	14	4	10	10	4	0	2	0
28.9	32.4	30.6	63	0	0	1	0	1	3	3	8	7	7	6	12	9	2	2	2	0
32.5	36.0	34.2	23	0	0	0	0	0	0	0	2	3	3	4	4	2	1	2	0	2
36.1	39.6	37.8	10	0	0	0	0	0	0	0	0	0	4	3	2	0	1	0	0	0
39.7	43.2	41.4	3	0	0	0	0	0	0	0	0	0	0	1	0	1	0	0	2	0
43.3	46.8	45.0	1	0	0	0	0	0	0	0	0	0	0	0	0	0	0	0	2	0
Total			353	2	6	15	24	46	35	25	32	32	35	21	31	24	11	4	8	2
**Average otolith weight**			**Age (years)**																	
**Ll**	**Ul**	**Midpoint**	**n**	**0** ^**+**^	**1**	**2**	**3**	**4**	**5**	**6**	**7**	**8**	**9**	**10**	**11**	**12**	**13**	**14**	**15**	**16**
0.021	0.090	0.05	40	2	6	12	9	10	0	0	1	0	0	0	0	0	0	0	0	0
0.091	0.160	0.12	38	0	0	1	8	11	8	3	3	3	1	0	0	0	0	0	0	0
0.161	0.220	0.19	57	0	0	1	5	19	14	8	6	2	1	0	1	0	0	0	0	0
0.221	0.290	0.26	6	0	0	0	0	1	2	0	1	1	0	1	0	0	0	0	0	0
0.291	0.360	0.33	117	0	0	0	2	5	11	14	18	21	22	5	10	5	2	0	2	0
0.361	0.430	0.39	55	0	0	0	0	0	0	0	2	3	7	9	10	13	8	0	3	0
0.431	0.490	0.46	29	0	0	0	0	0	0	0	0	2	4	5	9	4	1	2	2	0
0.491	0.560	0.53	4	0	0	0	0	0	0	0	0	0	0	1	1	0	0	2	0	0
0.561	0.630	0.60	6	0	0	0	0	0	0	0	1	0	0	0	0	2	1	0	0	2
0.631	0.700	0.66	1	0	0	0	0	0	0	0	0	0	0	0	0	0	0	0	1	0
Total			353	2	6	14	24	46	35	25	32	32	35	21	31	24	12	4	8	2

Lower (Ll) and upper (Ul) limits in total length (TL cm) and in average otolith weight (g) with number of individuals per age (years).

### Growth parameterization

**Fig 6:**
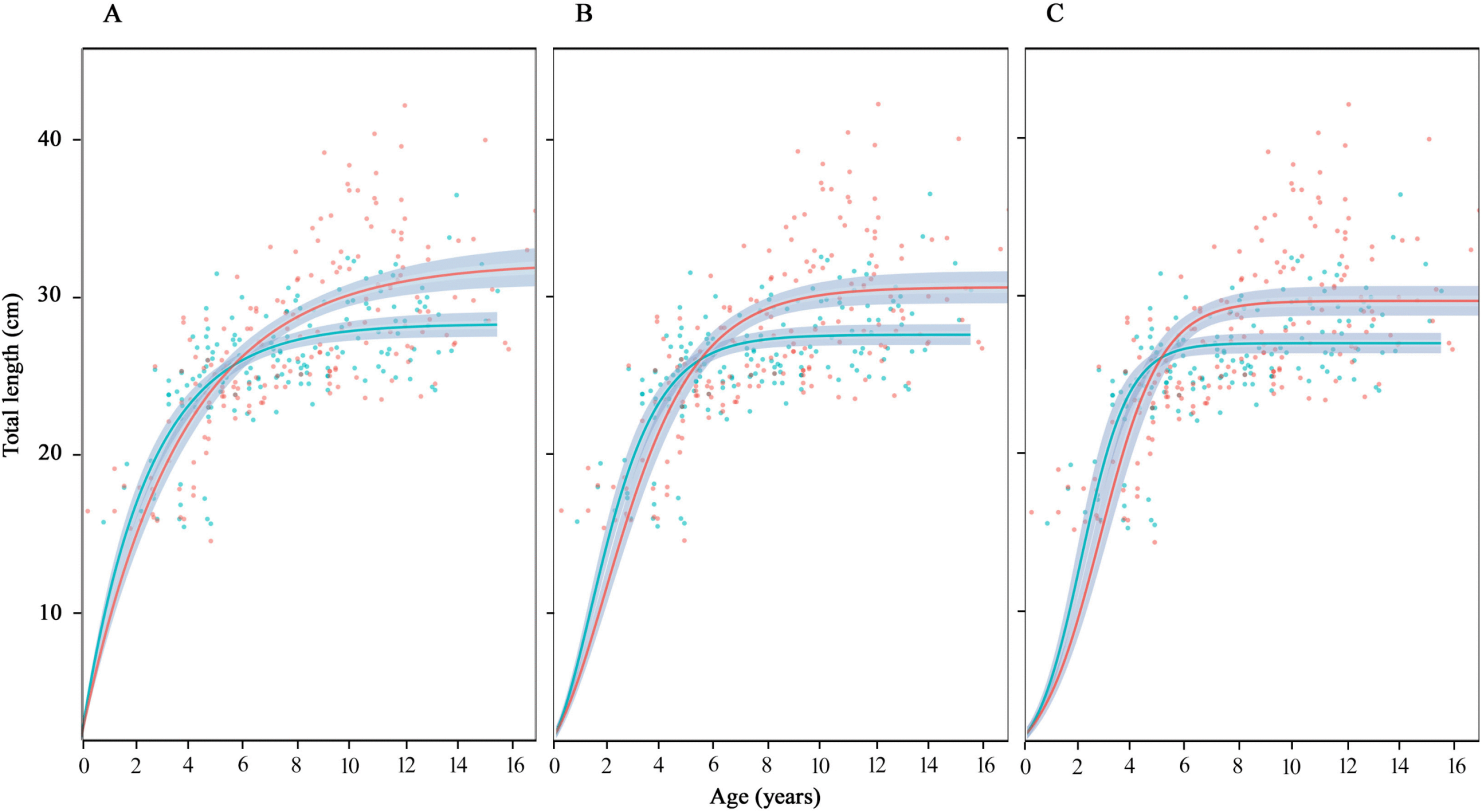
Comparison of growth curves by sex for *Lutjanus synagris* captured in southern Gulf of Mexico during 2008-2009. A– von Bertalanffy, B– Gompertz model, and C– logistic model. In red females and in blue males. Each growth model estimation includes in shadow the credibility intervals in gray (0.50, 0.95).

The Bayesian model selection LOOCV showed that VBGM displayed the best fit for the observed length at age for both sexes, with the lowest LOOIC and full model weight (Table 2). For females 𝐿_∞_ = 32.21 cm (SD = 0.74), *k*_*1*_ = 0.27 year^-1^ (SD = 0.02), *L*_*0*_ = 2.06 cm (SD = 0.20) and for males 𝐿_∞_ = 28.32 cm (SD = 0.40), *k*_*1*_ = 0.41 year^-1^ (SD = 0.03) and *L*_*0*_  = 2.03 cm (SD = 0.20) (Fig 6; Table 2). The credibility intervals of 𝐿_∞_, *k*_*1*_ and *L*_*0*_ (2.5%, 97.5%) did not overlapped in females (𝐿_∞_ = 30.83, 33.72, *k*_1_ = 0.23, 0.32, *L*_*0*_ *=* 1.66, 2.45) and males (𝐿_∞_ = 27.57, 29.15, *k*_1_ = 0.35, 0.47 and *L*_*0*_ *=* 1.64, 2.43) indicating a differential growth between sexes (Table 2). The $\emptyset^{\prime }$ values, calculated from the VBGM growth parameters, were $\emptyset^{\prime }$= 2.45 for females and $\emptyset^{\prime }$= 2.52 for males.

**Table 2:** Growth parameters and its statistical estimates for *Lutjanus synagris* captured in southern Gulf of Mexico during 2008–2009. Estimates were obtained by the Bayesian growth by sex. Model comparison is based on leave-one-cross-validation or leave-one-out-information-criterion.

	*L*_∞ (cm)_ mean±SD	2.5%, 97.5%	*k* (year^-1^) mean±SD	2.5%, 97.5%	*L*_*0*_ (cm) mean±SD	2.5%, 97.5%	σ mean±SD	2.5%, 97.5%	LOOIC	SE	ΔLOOIC	LOOICw
*Females*												
VBGM	32.21 ± 0.74	30.83, 33.72	0.27 ± 0.02	0.23, 0.32	2.06 ± 0.20	1.66, 2.45	4.18 ± 0.22	3.79, 4.64	1079	22.12	0.0	1.00
Gompertz	30.36 ± 0.49	29.49, 31.34	0.53 ± 0.03	0.47, 0.60	2.18 ± 0.23	1.80, 2.56	4.43 ± 0.23	4.01, 4.92	1101	22.67	22.0	0.00
Logistic	29.76 ± 0.48	28.84, 30.70	0.89 ± 0.06	0.78, 1.01	2.31 ± 0.19	1.93, 2.68	4.74 ± 0.25	4.73, 5.26	1127	22.53	48.0	0.00
*Males*												
VBGM	28.32 ± 0.40	27.57, 29.15	0.41 ± 0.03	0.35, 0.47	2.03 ± 0.20	1.64, 2.43	2.87 ± 0.16	2.50, 3.21	813.7	21.25	0.0	1.00
Gompertz	27.56 ± 0.34	26.89, 28.24	0.70 ± 0.04	0.62, 0.79	2.14 ± 0.20	1.75, 2.52	3.04 ± 0.17	3.04, 3.40	833.6	23.13	19.9	0.00
Logistic	27.10 ± 0.33	26.48, 27.75	1.14 ± 0.07	1.02, 1.30	2.25 ± 1.88	1.88, 2.63	3.25 ± 0.19	3.24, 3.64	855.8	25.08	42.1	0.00

$L_{\infty }$𝐿_∞_ = maximum mean length, for VBGM *k*_1_ *=* growth coefficient as rate at which $L_{\infty }$ is reached, for Gompertz *k*_2_ = exponential rate of decrease in relative growth with age and for Logistic *k*_3_ = relative growth rate parameter, *L*_*0*_ = length at age zero and σ = observation variability. Mean = values are posterior means, SD = standard deviations, credible intervals = 2.5%, 97.5%. All R̂ ≈ 1 indicating convergence. ΔLOOIC = difference relative to best-fitting model. LOOICw = weights.

### Life history

**Fig 7:**
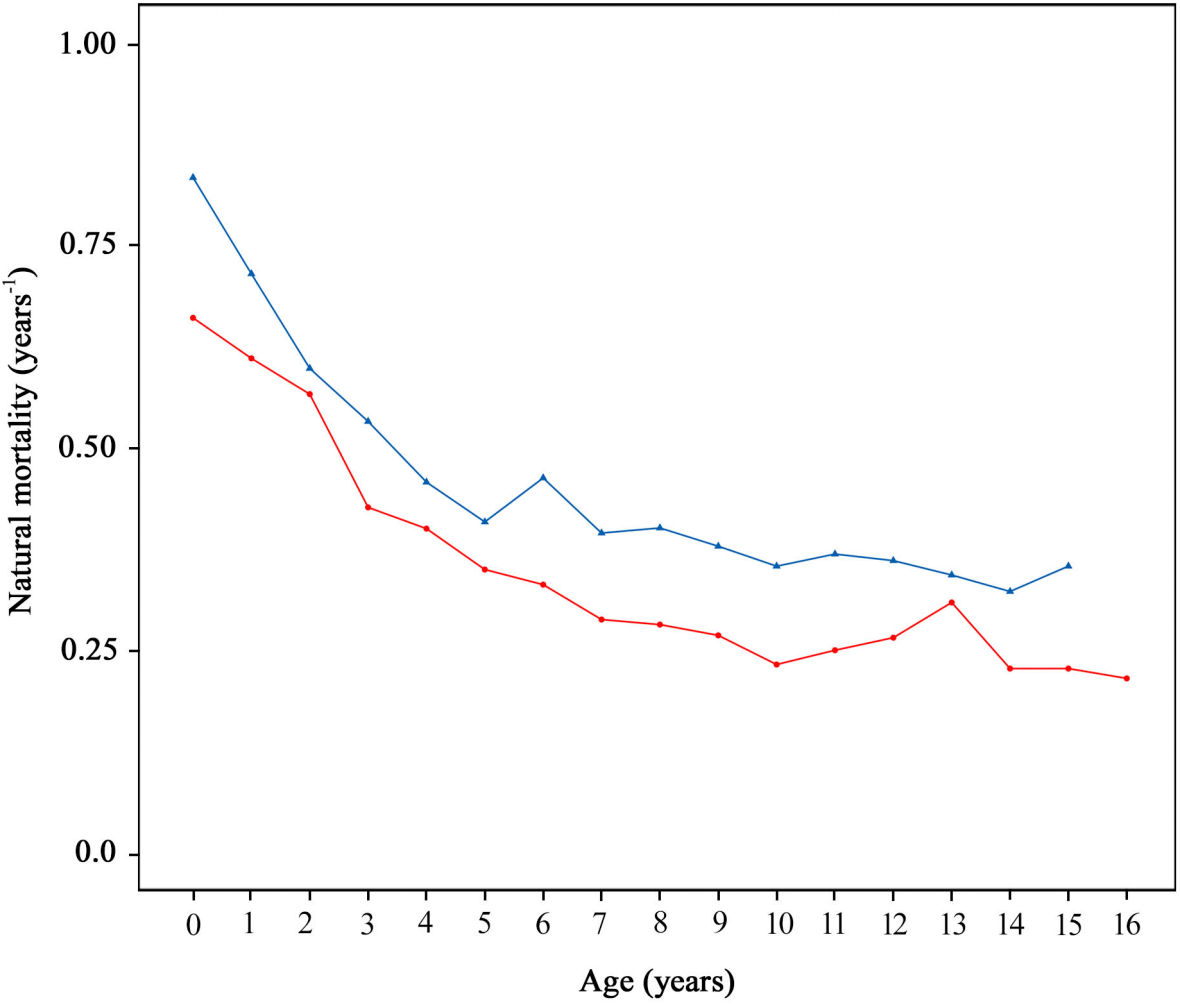
Age-based natural mortality by sex of *Lutjanus synagris* captured in southern Gulf of Mexico during 2008–2009. In red females and in blue males.

Overall natural mortality (M_overall_) was lower in females (M_overall_ = 0.39 year^-1^) than in males (M_overall_ = 0.41 year^-1^). Mortality-at-age values exhibited an exponential decline from very young individuals (0^+^): M = 0.66 year^-1^ for females and 0.83 year^-1^ for males, to 1 and 5 years (females, M = 0.35 year^-1^; males, M = 0.41 year^-1^), and continued to decrease until 10 years (females, M = 0.23 year^-1^; males, M = 0.36 year^-1^). The decline in M-at-age versus individual age was consistently lesser in females, reaching its lowest value (M = 0.22 year^-1^) at 16 years, than in males at 15 years (M = 0.36 year^-1^) (Fig 7).

**Fig 8:**
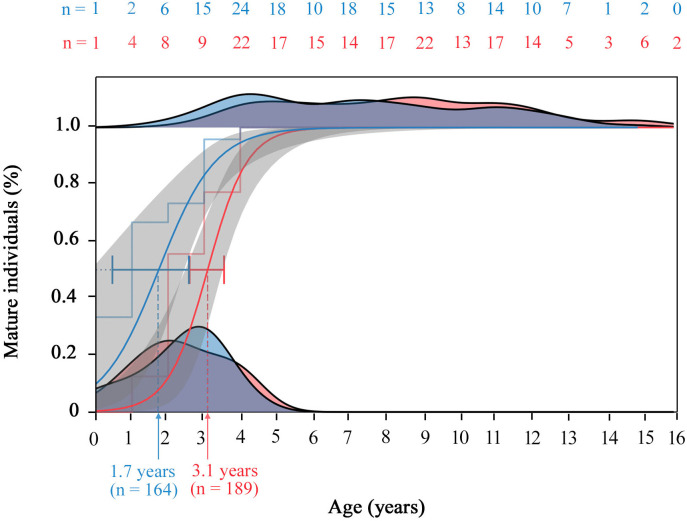
Age at sexual maturity (*A*_50_) by sex of *Lutjanus synagris* captured in southern Gulf of Mexico during 2008–2009. Distributions of immature (0) and mature individuals (1) analyzed with logistic general lineal function (continuous lines) their standard error (grey shading). Age at maturity (A_50_) (dotted lines) and their 95% confidence intervals (horizontal error bars) in years by sex. In red females and in blue males.

The southern GoM lane snapper population exhibited sex-differentiated age at sexual maturity. Males matured at an earlier age (A_50_ = 1.68 years; 95% CI = 0.47–2.34 years) than females (A_50_ = 3.11 years; 95% CI = 2.61–3.54 years) (Fig 8). All individuals of both sexes were mature at 6 years of age (Fig 8). The youngest mature male had an A_min_ = 1 year old (19.40 cm TL), and the youngest mature female had an A_min_ = 2 years old (25.60 cm TL).

**Fig 9:**
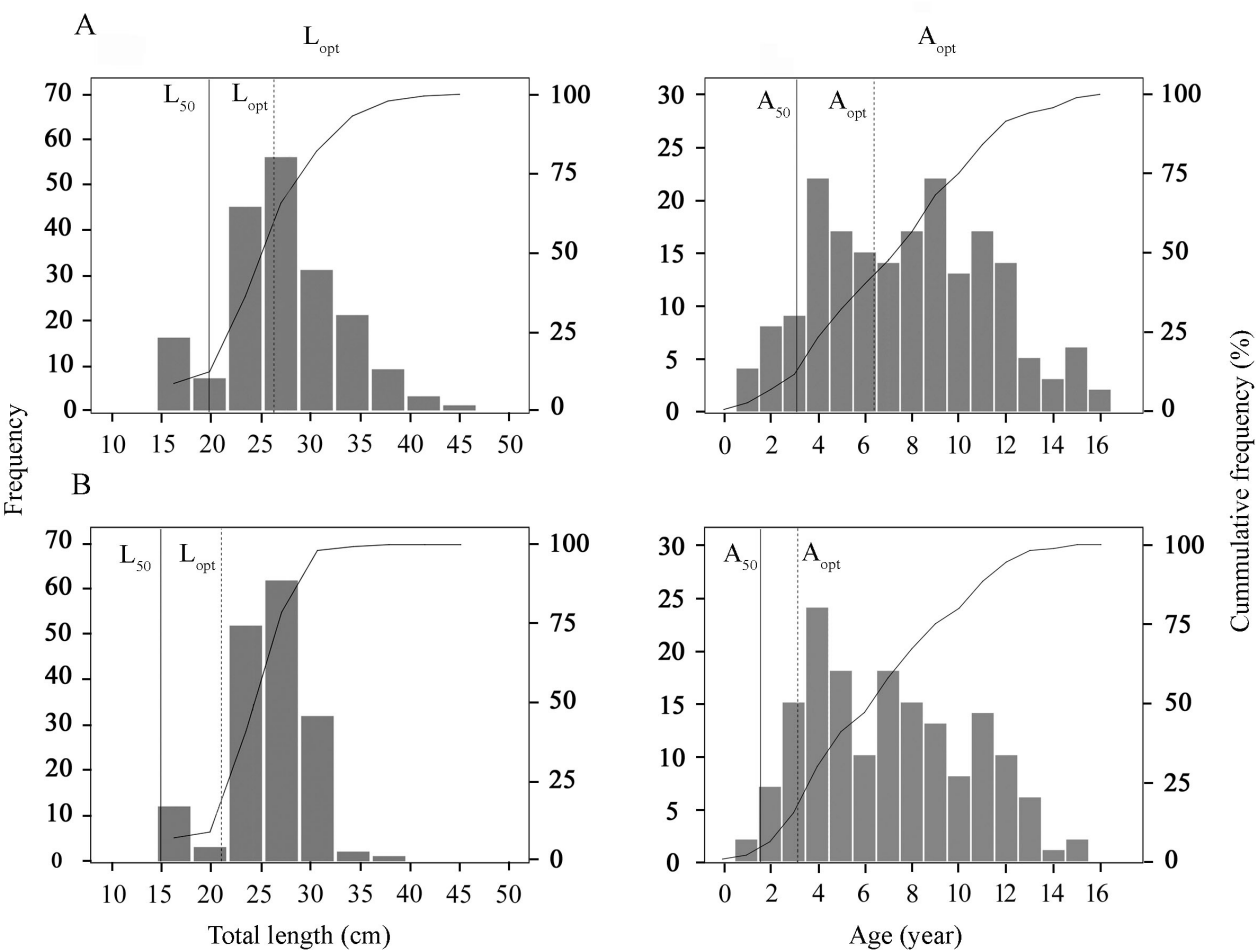
Optimal length and optimal age of *Lutjanus synagris* captured in southern Gulf of Mexico during 2008–2009. A– females and B– males. Frequency distributions of the number of individuals (bars), cumulative frequency distributions of length and age (continuous grey line), L_50_ and A_50_ (vertical continuous line by sex), mean optimal length (L_opt_) and mean optimal age A_opt_ (vertical dotted line by sex).

Females had higher *L*_*opt*_ (26.17 cm TL) and *A*_*opt*_ (6.20 years) values (Fig 9A) than males (*L*_*opt*_ = 21.24 cm TL; *A*_*opt*_ = 3.38 years) (Fig 9B).

## Discussion

The present study provides the first age-based life history characterization of lane snapper from southern GoM using otolith thin-sections combined with a multi-model approach to determine growth. The results indicated marked sexual dimorphism where males reach age at maturity earlier than females, exhibit faster growth, higher natural mortality and shorter lifespan. Since sampling was performed 17 years ago, the present results could be considered as historical baselines.

Lane snapper age has been determined in populations from the northern and southern GoM, Florida, Bermuda, Jamaica, Guatemala, and Brazil. Wide variation in individual size at a given age has been reported in this study and all other. This occurs independently of the methodological approach, differences in the prevailing environmental conditions or the fishing gears used in sampling [9,18,24,44]. Considering only those age studies that used otolith thin sections, including the present one, observations on variation in individual size at age are consistent. Lane snapper from Florida showed length at age variations of 10–20 cm TL for ages 6–10 years [24], while in Jamaica variation was observed of up to 24 cm FL (range: 15–39 cm) in individuals aged 14 years [9], and in the present study, length variation of approximately 18 cm for individuals aged 4–6 years and 21 cm in individuals with 10–13 years. Size at age variations have also been reported in other snappers such as yellowtail snapper [23,81,82], northern red snapper [83,84], and gray snapper [85].

Due to the variability in length at age in snappers, it is inadvisable to use length-frequency distributions to estimate age beyond the first few years [62], so the use of OW as a descriptor to age could be an alternative. In this study OW had a stronger relationship with age, than TL, which would allow more accurate age estimations [5,86]. Otolith weight has been used alternatively and supplementary to traditional age estimation over the last two decades [87]. Faster growing fish have a higher amount of protein in their otoliths resulting on lighter otoliths, while slow growing fish deposits a high proportion of calcium carbonate resulting in heavier otoliths [88]. But the most useful otolith characteristic to age studies is that OW continues to increase with age unlike other variables (fish length, fish weight, otolith length) [86,89]. In extreme cases if fish growth ceases, the increment of otolith growth will continue uncoupling to the somatic growth [90].

High variability in length at age could be related to the protracted spawning season of the species, where sexually active females and males were observed from March to July [42]. Individuals born earlier at the beginning of the reproductive season may have longer time to growth becoming bigger than the ones born later in the season. To reduce the possibility that variability in length at age is caused by errors in annuli reading precision, careful considerations are required, as age determination in Lutjanids is challenging. First, the detection of the first annulus is crucial, since in Lutjanids there are checks or false rings (from 1 to 3) near the otolith core [62,91] and the presence of hyaline (translucent) bands, produce by a rapid temperature increase, may mimic the first annulus [92]. These could explain the higher values of APE recorded for younger individuals than older.

Second, the validation, for example via MIA, of the frequency and period of formation of the annulus. In lane snapper form southern GoM, growth rings form annually during May and June, consistent with previous studies using MIA for age determination: April to June in Cuban populations [17]; April to September in the northern GoM [13]; May and August in Trinidad, West Indies [18]; July in Jamaica [9]; April and May in Guatemala [21]; and April to June in Bermuda [5]. This temporal OZ formation patterns in lane snapper varied by age, which has been reported in other snappers and fishes [62,63,93]. Variations in annulus deposition period respond in part to environmental conditions and breeding seasons. In the tropics, reproductive activity influences otolith calcification continuity and efficiency, since spawning-associated physiological rhythms are responsible for growth mark formation [18,94]. This is corroborated with the results in southern GoM lane snapper, where the annulus formation coincided with its March to July breeding season [42].

Lastly, in many fishes there is a variation between apexes on otolith thin sections, an artifact of morphological asymmetry where all growth rings are visible along a single otolith axis [95,96]. This variation between apexes may be due to metabolic responses produced by changes in diet, environmental conditions, or slow growth, all resulting in crammed annuli that make ring identification and counting more challenging in one apex over the other [94,97–99]. To control for potential errors in ring counts, we recommend that counts should be done near the crest of the sulcus acusticus and on the ventral apex (where all rings were visible), specially for older individuals [62,100].

Lane snapper clearly grows rapidly during the first year of life, with growth rate progressively declining with proximity to asymptotic size (approximately 11 years in females, and 8 years in males). The maximum age observed in the present study was a 16-year-old female similar to that reported in other areas of the species’ geographic distribution: 17 years for females in Florida [13], 18 years for both sexes in Brazil [22], and 19 years in Bermuda [5]. Maximum size was also larger in females than in males. This sexual dimorphism is consistent with that reported for the Guatemalan population [21], and contrary to the populations in northern GoM and Trinidad, West Indies, where males had larger maximum sizes although the longest-lived individuals were always females [13,18]. In southern GoM lane snapper males reach asymptotic length more quickly than females. This is similar to populations from Florida, Jamaica and Guatemala (Caribbean Sea) [9,13,21]. In contrast, the Bermuda and Brazil populations exhibited no sexual differentiation in growth [5,22].

Sex-growth differences may be related to environmental factors, mainly temperature, water quality, genetic and epigenetic regulation, species-specific life history strategies [21,101–104] or even be an evolutionary response to selective fishing pressure exerted on one of the sexes [85,105]. Females in many species tend to exhibit greater growth and larger size than males, often associated with delayed maturation [103,106]. Our A_50_ results are consistent with the L_50_ data (19.25 cm TL for females, 14.73 cm TL for males) reported previously for the species [42]. The age at sexual maturity values for southern GoM lane snapper were similar to those reported for populations in Guatemala (A_50_ = 2.40 years for males, 3.50 years for females) [21] and in Brazil (A_50_ = 2.80 years for both sexes) [22]. Early maturation in males beginning prior to one year of age in approximately 20–24% of them [2,9,18] is also reported in populations of Guatemala and Trinidad, West Indies [18,21]. Inter-population differences in size-at-maturity respond to depth, habitat type, and food availability, which in consequence influence energy balance, growth, and reproduction [107]. Analysing growth separately by sex helps to understand possible evolutionary responses to scenarios of overfishing and/or fluctuating climatic conditions, since both factors can affect age and size-at-maturity [108].

**Fig 10:**
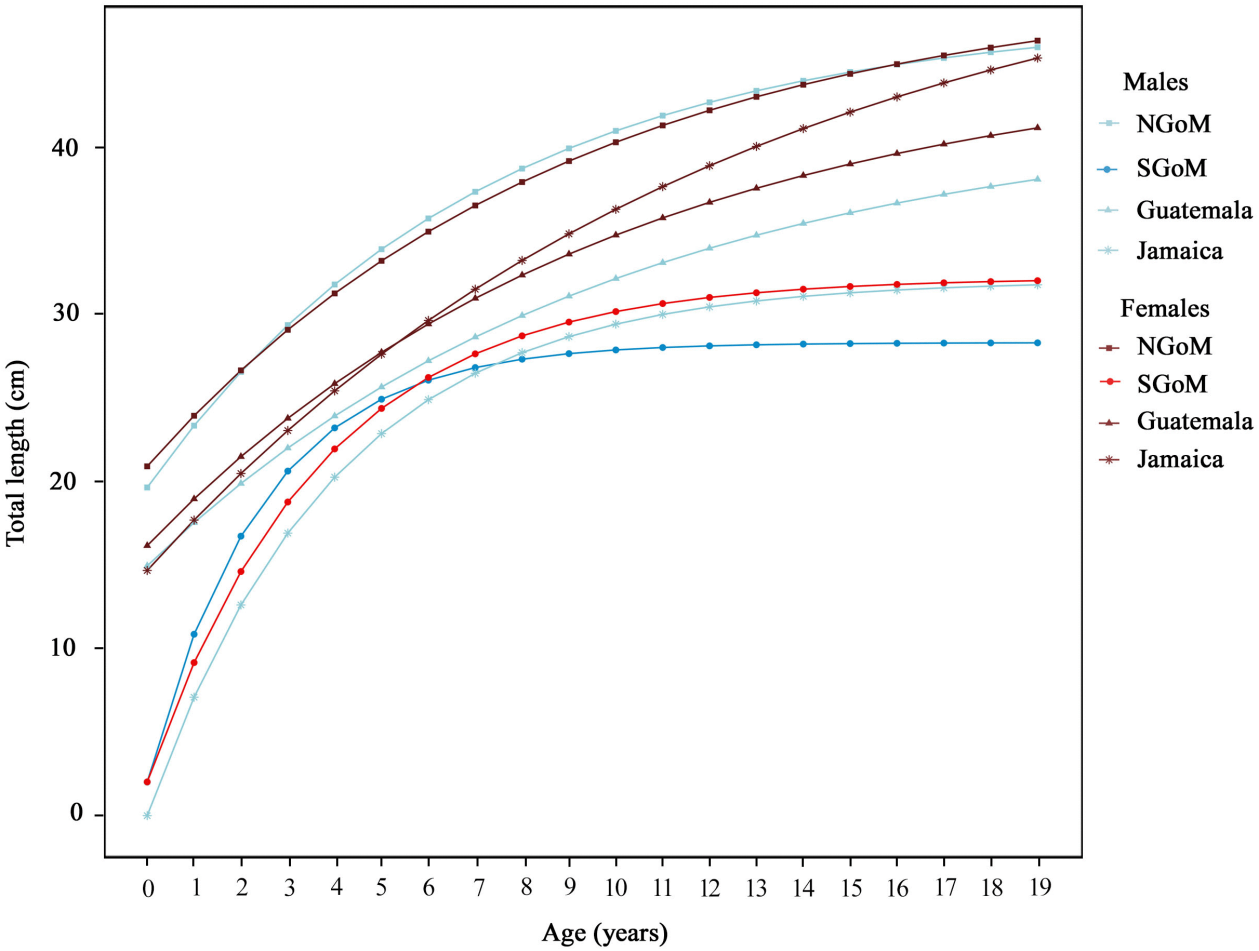
Sex-specific growth curves of *Lutjanus synagris* modeled using the von Bertalanffy growth model across its distribution area. Northern Gulf of Mexico (NGoM) [13]; Southern Gulf of Mexico (SGoM) (Present study); Guatemala [21]; Jamaica [9].

The VBGM model had the best fit to the data for both females and males. Previous studies that have evaluated growth in some snappers using a multi-model approach found different models to have the best fit for different species: logarithmic model for yellowtail snapper [30], and the VBGM for northern red snapper, gray snapper and mutton snapper [26,29,31]. Earlier research on growth in lane snapper based on otolith thin sections used only the VBGM, without testing its efficacy [5,13,22,23]. The VBGM does include terms representing the metabolic properties of assimilation, but changing environmental factors can cause growth patterns to deviate from ideal growth forms, making the model unsuitable for describing the first year of life in various species [109], therefore its fitness for the data should always be tested. The Ø’ values observed here (males Ø’ = 2.52; females Ø’ = 2.45) were within the range reported in other populations (Ø’ = 2.25–2.53). In other words, growth was similar between populations, save for those inhabiting temperate waters such as in Bermuda (Ø’ = 2.63; [5], and Brazil (Ø’ = 2.84, [22] (Fig 10, Table 3).

**Table 3:** Comparative age-based life history traits from sagittal otolith thin sections studies of *Lutjanus synagris* populations with von Bertalanffy growth model.

Region	${𝐋}_{\infty }$ (cm)	k_1_ (y^-1^)	Φ’	A_max_ (y)	t_max_ (y)	M (y^-1^)	A_50_ (y)	Reference
***Combined sexes***								
NGoM	50.10 LT	0.13	2.53	10	21.59	0.40		[24]
NGoM	47.93 LT	0.13	2.46	17	18.83	0.23		[13]
NGoM	29.62 LT	0.24	2.32		7.98			[23]
Bermuda	33.10 LF	0.39	2.63	19	5.74			[5]
Brazil	56.00 LT	0.22	2.84	18	13.64	0.28–0.36	2.80	[22]
***Females***								
NGoM	50.04 LT	0.11	2.42	17	22.35	0.24		[13]
SGoM	32.21 LT	0.27	2.45	16	10.87	0.39	3.37	Present study
Guatemala	45.58 LT	0.10	2.32	11	25.62	0.27	3.50	[21]
Jamaica	53.87 LF	0.08	2.34	14	33.53			[9]
***Males***								
NGoM	47.9 LT	0.14	2.50	11	17.67	0.21		[13]
SGoM	28.32 LT	0.41	2.52	15	7.40	0.41	1.79	Present study
Guatemala	42.17 LT	0.10	2.25	11	25.62	0.27	2.40	[21]
Jamaica	32.00 LF	0.25	2.41		12.00			[9]

L_∞_ = asymptotic length, k_1_ = growth coefficient, A_max_ = maximum age reported, t_max_ = longevity, Ø’ = growth performance index, M = natural mortality, TL = total length, FL = fork length, NGoM = northern Gulf of Mexico; SGoM = southern Gulf of Mexico.

The sexual dimorphism observed in the somatic growth of lane snapper and the age-at- maturity impact growth rates, longevity, and natural mortality values [110]. For natural mortality, values were 0.39 year^-1^ for females and 0.41 year^-1^ for males, within the range of the M values reported for the specie in Florida, Guatemala, NGoM, and Brazil populations (M = 0.21–0.40 year^-1^) (Table 3) [13,21,22,24]. Natural mortality varies between populations in relation to predation and food availability [111], environmental conditions [112], life-history traits [113], and even human activities such as oil spills [114].

Assessing the potential impact of fishing on each lane snapper sex is essential, considering that the growth characteristics of males could make them more vulnerable to fishing. One way to address sexual dimorphism due to growth is to determine optimal size and age at capture (L_opt_ and A_opt_) for lane snapper. These calculations are based on the growth and M values and represent a key parameter in regulating lane snapper exploitation [22]. Both values can be expected to be higher than L_50_ [80]. For any commercial species, management measures used to maximize fishing yield, should consider a catch size corresponding to L_opt_ ± 10% [80]. Due to the sexual differences in life parameter values in the southern GoM lane snapper population, minimum capture size should be based on the L_opt_ value of females (26.17 cm TL), with an average A_opt_ of 6.20 years of age. This criterion would simultaneously protect males, which reach L_opt_ (21.24 cm TL) at an average A_opt_ of 3.38 years of age. This would protect most immature individuals by preventing overfishing of younger individuals, and the smallest mature males, in the southern GoM lane snapper population. Currently most lane snapper captured in southern GoM display sizes ranging from 17–43 cm FL with a mean size of 26.0 ± 2.9 cm FL [33], so there would almost be no resistance from fishermen to the imposition of a minimum legal size of capture of ± 26 cm TL.

This study provides the first robust age-based life history assessment for lane snapper in the southern Gulf of Mexico, demonstrating clear sexual differences in growth, maturation, longevity, and mortality. Importantly, the identification of sex-specific maturity and optimal harvest sizes/ages underscores the need for management measures based on the most conservative reference points, particularly those of females. These findings provide critical inputs for stock assessment of the population over time and are vital to developing the foundations of urgent regulatory guidelines promoting population recovery and sustainability of the resource.

## Supporting information

S1 Fig Graphs for the estimation of the growth parameters obtained by the Bayesian growth method using MCMC, for growth model selection. Upper: Females, Lower: Males. A- von Bertalanffy growth model, B- Gompertz model, C- Logistic model.
(TIF)

S3 Fig Bootstrap-based 95% confidence intervals for total length differences in quantiles Q10 = −4.12–0.74, Q25 = −0.40–0.50, Q50 = −0.10–1.00, Q75 = 0.40–1.60, Q90 = 0.20–2.50) between the subsample (dotted line) and all-dataset individuals (solid line) of *Lutjanus synagris* captured during 2008–2009. (TIF)

S2 Data Data set of *Lutjanus synagris* in the southern Gulf of Mexico captured in southern Gulf of Mexico during 2008–2009. Total fish length and weight, mean otolith weight (sagittae), age, sex and sexual maturity variables used to obtained growth parameters, age at maturity, longevity and mortality. (XLSX)

S4 Fig Linear relationships of *Lutjanus synagris* in the southern Gulf of Mexico captured in southern Gulf of Mexico during 2008–2009. **A– total length and age, B– otolith weight and age.** Age (dots) and regression line (continuous line) with confidence intervals (95%) grey area of the line. Color dots represent the otolith weight classes of the subsample.
(PDF)
