# Supplementary figures and images for: Lutjanus synagris (Linnaeus 1758) age-based life history using a multi-model inference approach for growth in the southern Gulf of Mexico
Source: PLoS One. 2026 Jul 21;21(7):e0353946. doi: 10.1371/journal.pone.0353946 (PMC13387562; doi:10.1371/journal.pone.0353946)

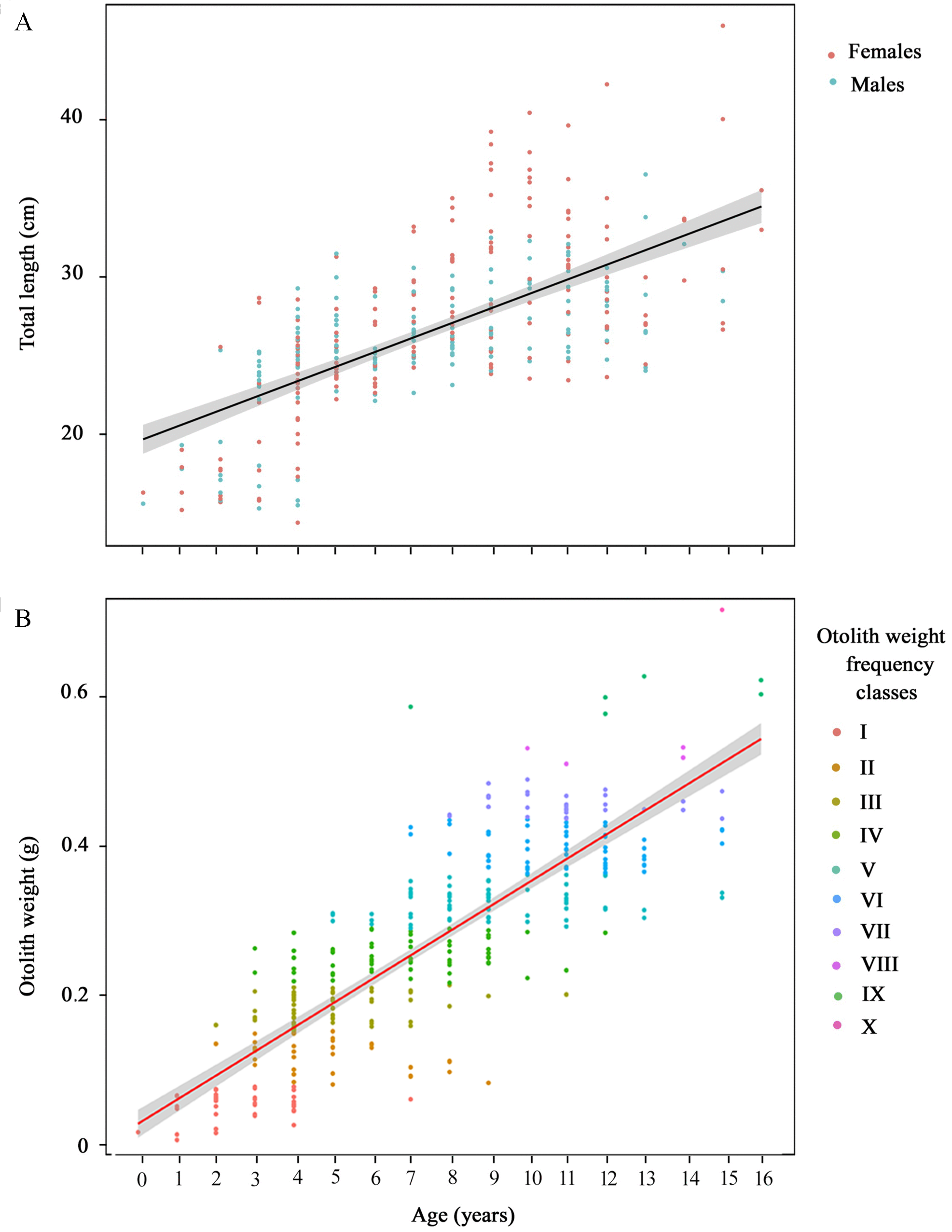

Supplement: S3 Fig — (TIF) [file pone.0353946.s002.tif]

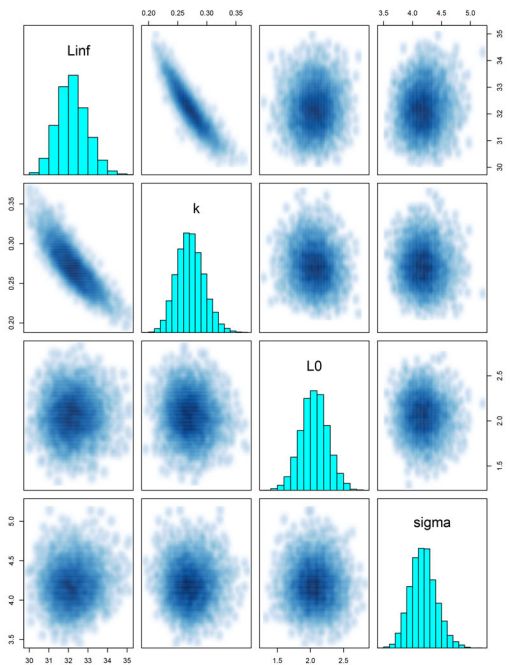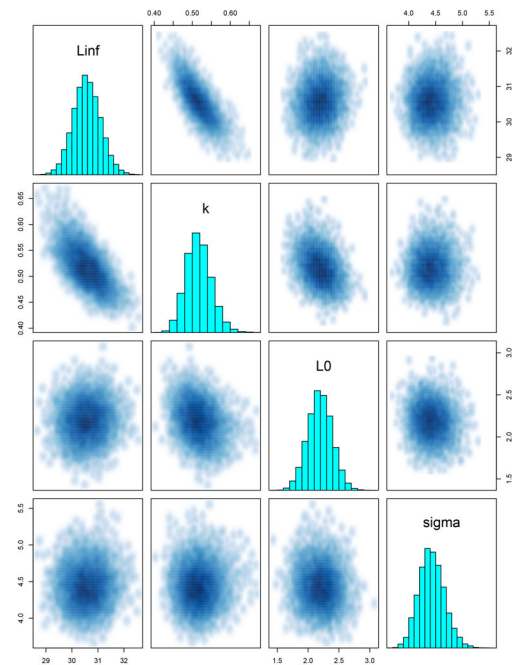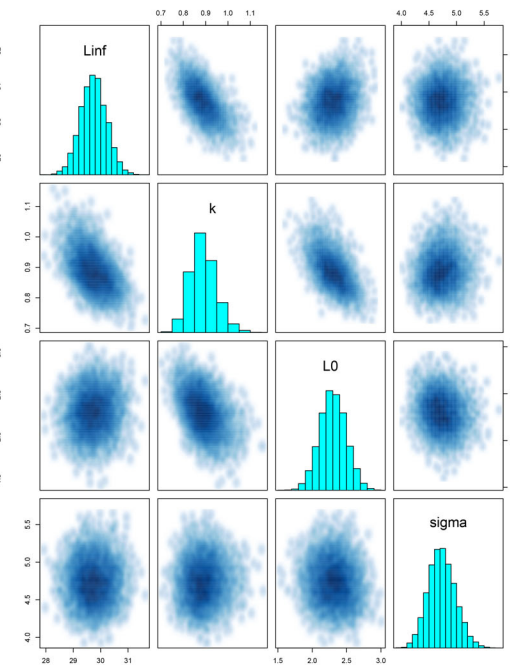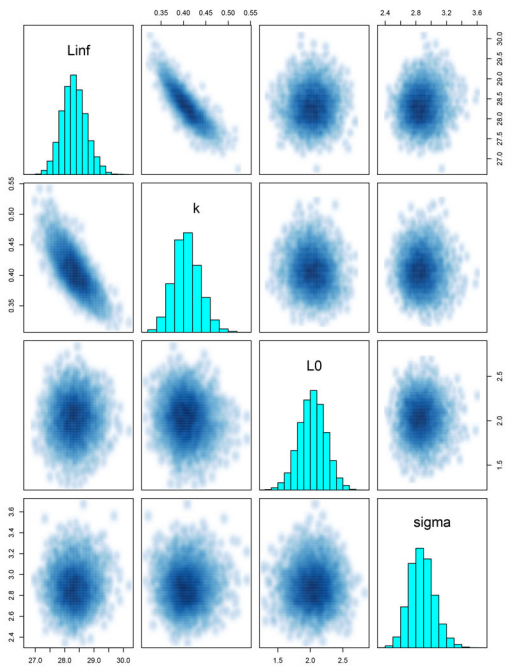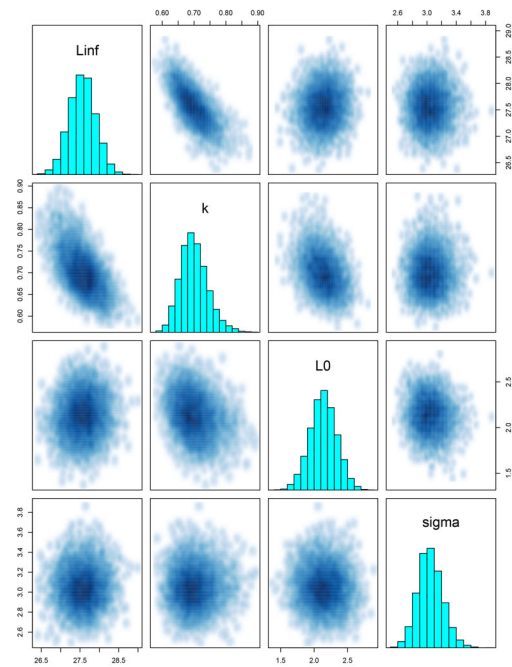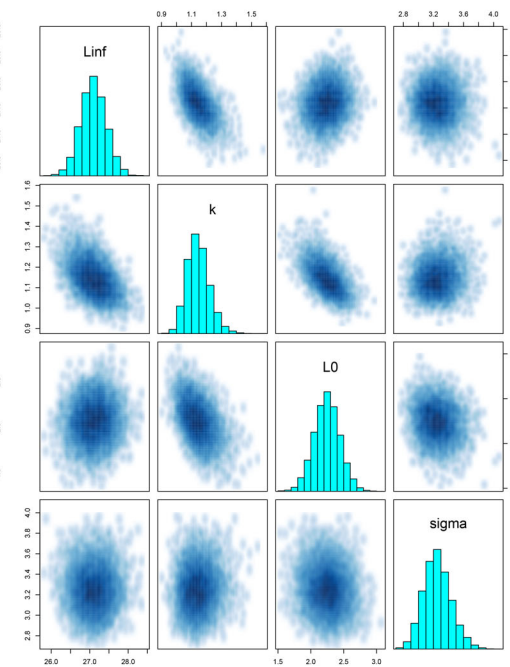

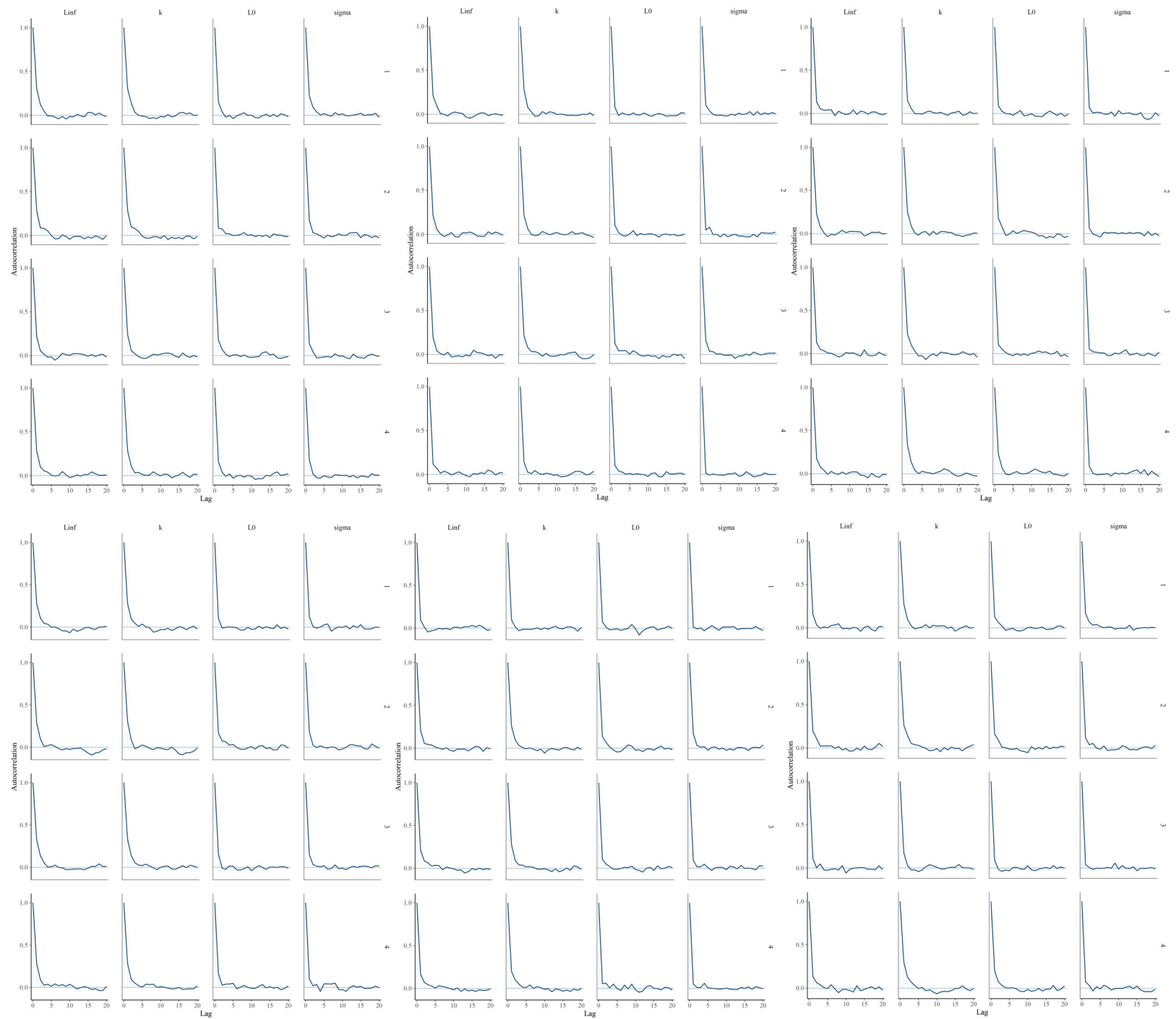

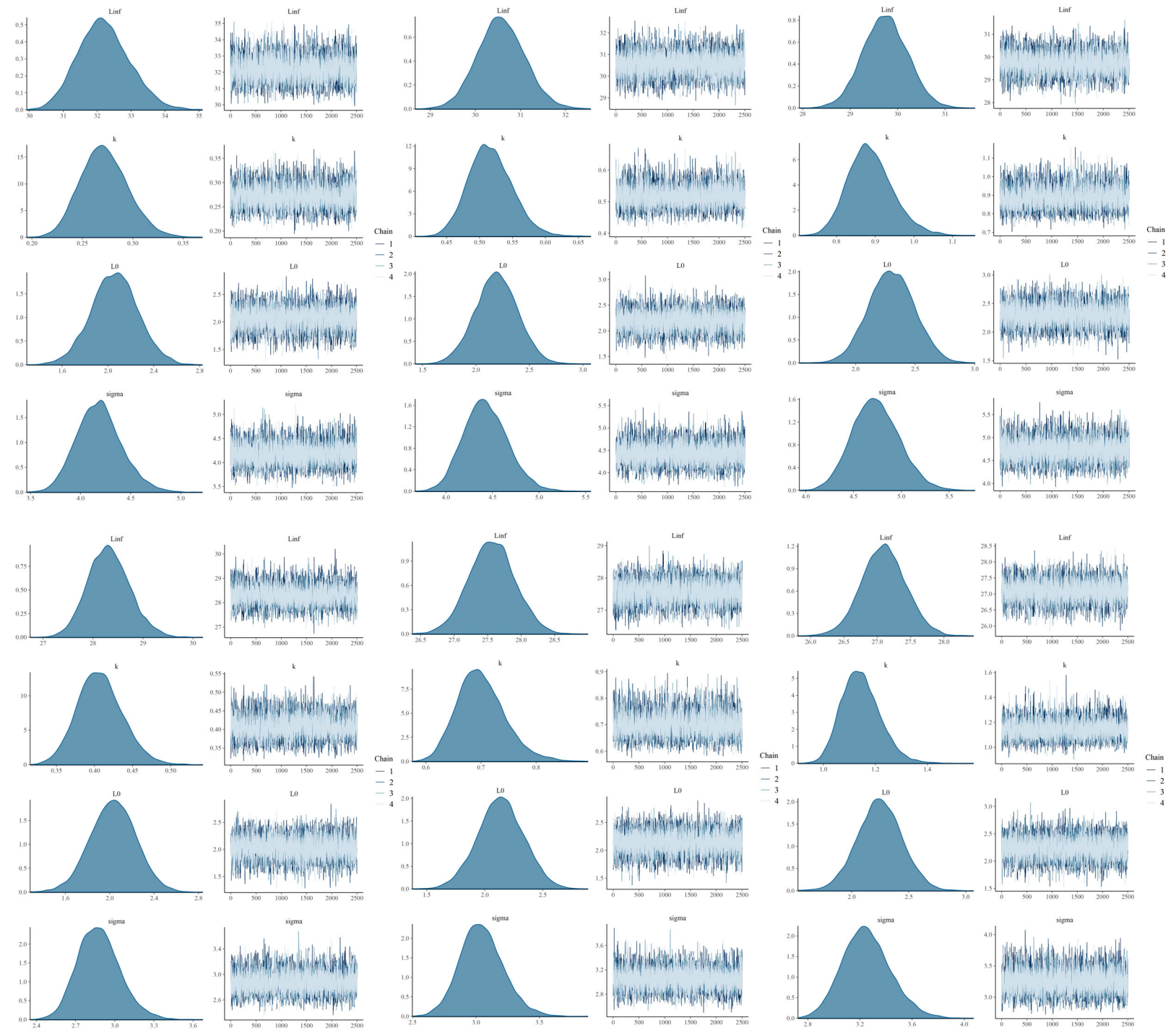

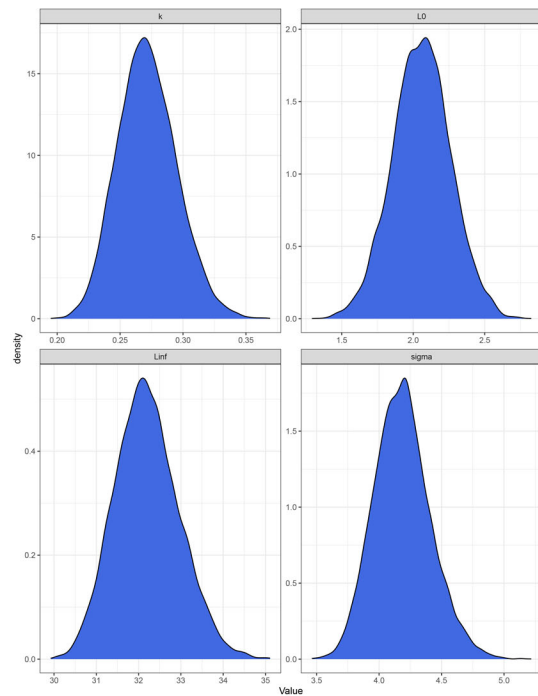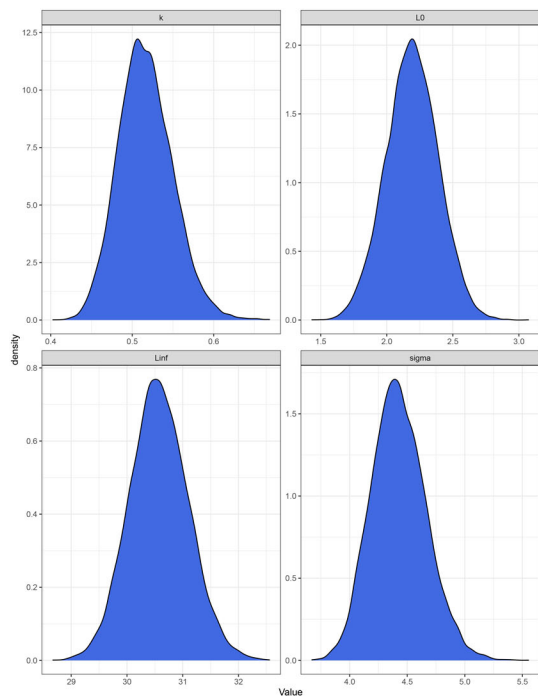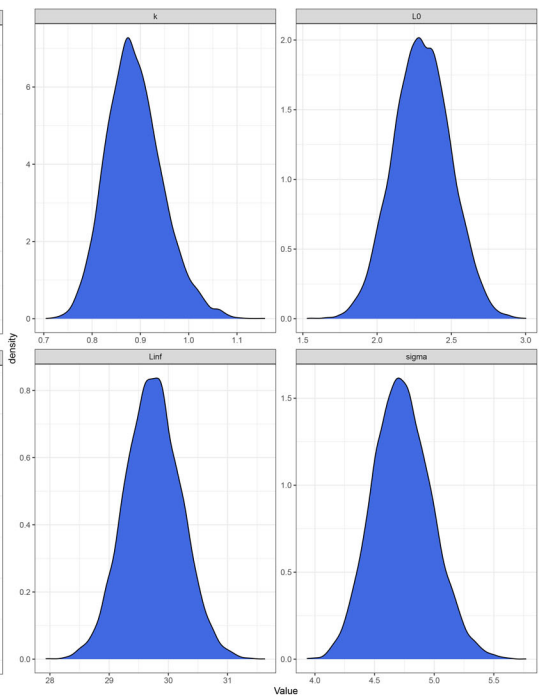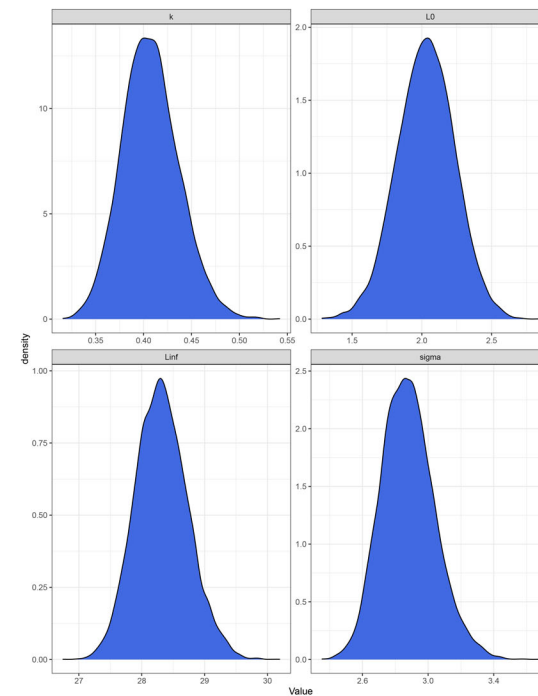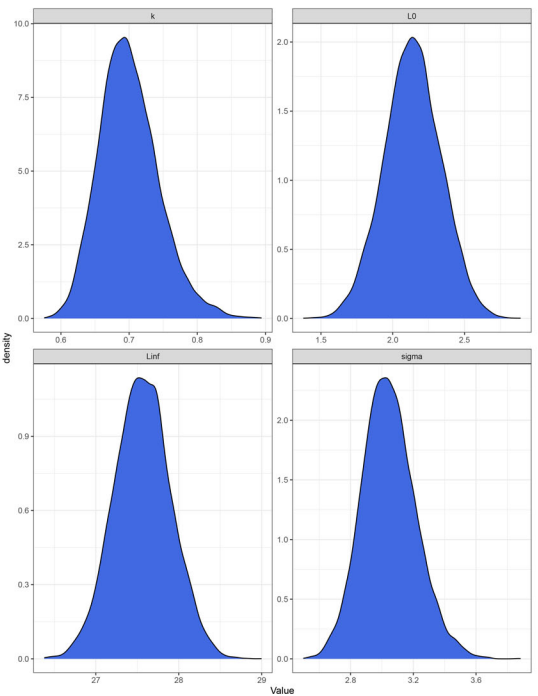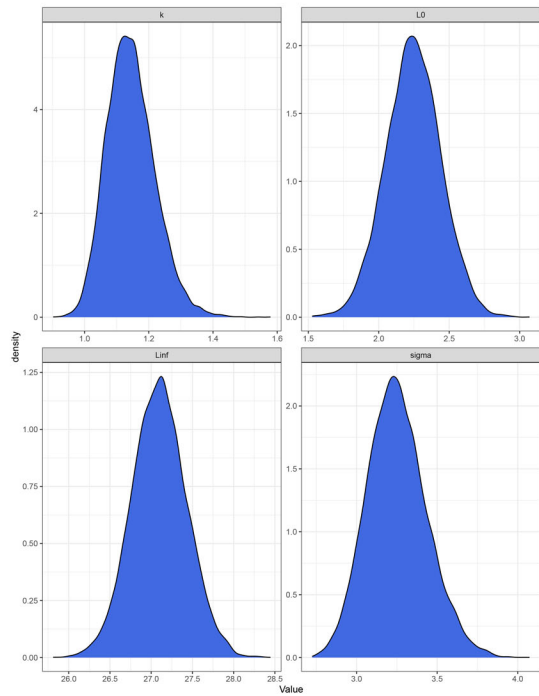

Supplement: S4 Fig — A– total length and age, B– otolith weight and age. Age (dots) and regression line (continuous line) with confidence intervals (95%) grey area of the line. Color dots represent the otolith weight classes of the subsample. (PDF) [file pone.0353946.s004.pdf]
